# Allochthonous marsh subsidies enhances food web productivity in an estuary and its surrounding ecosystem mosaic

**DOI:** 10.1371/journal.pone.0296836

**Published:** 2024-02-29

**Authors:** Melanie J. Davis, Isa Woo, Susan E. W. De La Cruz, Christopher S. Ellings, Sayre Hodgson, Glynnis Nakai

**Affiliations:** 1 U.S. Geological Survey, Western Ecological Research Center, Olympia Substation, Olympia, Washington, United States of America; 2 U.S. Geological Survey, Western Ecological Research Center, San Francisco Bay Estuary Field Station, Moffett Field, California, United States of America; 3 Nisqually Indian Tribe, Department of Natural Resources, Olympia, Washington, United States of America; 4 U.S. Fish and Wildlife Service, Billy Frank Jr. Nisqually National Wildlife Refuge, Olympia, Washington, United States of America; CIFRI: Central Inland Fisheries Research Institute, INDIA

## Abstract

Terrestrial organic matter is believed to play an important role in promoting resilient estuarine food webs, but the inherent interconnectivity of estuarine systems often obscures the origins and importance of these terrestrial inputs. To determine the relative contributions of terrestrial (allochthonous) and aquatic (autochthonous) organic matter to the estuarine food web, we analyzed carbon, nitrogen, and sulfur stable isotopes from multiple trophic levels, environmental strata, and habitats throughout the estuarine habitat mosaic. We used a Bayesian stable isotope mixing model (SIMM) to parse out relationships among primary producers, invertebrates, and a pelagic and demersal fish species (juvenile Chinook salmon and sculpin, respectively). The study was carried out in the Nisqually River Delta (NRD), Washington, USA, a recently-restored, macrotidal estuary with a diverse habitat mosaic. Plant groupings of macroalgae, eelgrass, and tidal marsh plants served as the primary base components of the NRD food web. About 90% of demersal sculpin diets were comprised of benthic and pelagic crustaceans that were fed by autochthonous organic matter contributions from aquatic vegetation. Juvenile salmon, on the other hand, derived their energy from a mix of terrestrial, pelagic, and benthic prey, including insects, dipterans, and crustaceans. Consequently, allochthonous terrestrial contributions of organic matter were much greater for salmon, ranging between 26 and 43%. These findings demonstrate how connectivity among estuarine habitat types and environmental strata facilitates organic matter subsidies. This suggests that management actions that improve or restore lateral habitat connectivity as well as terrestrial-aquatic linkages may enhance allochthonous subsidies, promoting increased prey resources and ecosystem benefits in estuaries.

## Introduction

Connectivity is a hallmark feature of ecological systems, allowing for flows of energy, nutrients, and species across diverse landscapes, and conferring resilience in the face of disturbance and habitat change [1–5]. Connectivity between terrestrial and aquatic systems is particularly important because the reciprocal exchange of organic matter and energy can subsidize food webs and promote enhanced productivity [6–10]. Estuaries are one example of a system where these terrestrial-aquatic linkages are a crucial component of ecosystem functioning. Estuaries are comprised of a mosaic of interconnected habitats with varying levels of tidal and riverine influence, all of which synergistically support fish and wildlife species and their respective prey resources [11–16]. Resources are distributed across terrestrial, pelagic, and benthic environmental strata, and the degree of exchange between each habitat type depends on its structural characteristics [17–20]. For example, features such as the type and density of adjacent vegetation, the frequency and duration of flooding, and a habitat’s proximity to other vegetated habitats can all influence terrestrial inputs to estuarine food webs.

Contributions of externally-sourced (allochthonous) and internally-sourced (autochthonous) organic matter form the base of the aquatic food web [21, 22]. In estuarine research, the terms “allochthonous” and “autochthonous” have been ascribed different meanings depending on the context. For example, Howe and Simenstad [17] referred to any marsh-derived organic material as autochthonous and riverine and marine phytoplankton as allochthonous because it originated from outside of their target study system. Conversely, Bouaziz et al. [23] defined phytoplankton and aquatic macrophytes as autochthonous and any organic matter of terrestrial origin as allochthonous. For the sake of our study, we define allochthonous sources of organic matter as terrestrial in origin and autochthonous sources as aquatic in origin [21, 22]. Allochthonous organic matter can come directly from terrestrial detritus (i.e. riparian leaves) making its way into the water column or via indirect consumptive pathways when primary consumers eat emergent tidal marsh plants and are subsequently consumed by predators. Autochthonous organic matter is derived from aquatic and epibenthic primary producers such as phytoplankton, microphytobenthos, macroalgae, and submerged aquatic vegetation [21, 24–26]. Both sources are associated with varying levels of riverine, marsh, and marine (or delta) influence [27]. Thus, estuarine food web subsidies occur not only along a terrestrial-aquatic gradient, but also along a salinity-habitat gradient.

Aquatic food webs are supported by varying basal resources and function differently depending on whether the base of the food web is dominated by allochthonous or autochthonous organic matter [28–31]. Allochthonous organic matter contributes significantly to food webs in many aquatic systems, including estuaries, but relative contributions can vary widely depending on geographic location and climate [17, 32–34]. A shift from terrestrial to aquatic food web pathways can bolster productivity, resulting in subsequent enhancements to carrying capacity and fish consumer growth [35–37]; however, a food web based entirely on autochthonous pathways may be less resilient to perturbations and detrimental environmental conditions such as nutrient imbalances, elevated temperatures, lower dissolved oxygen levels, and poor prey quality [36, 38–40]. The inherent interconnectivity of estuarine systems makes it difficult to parse out the relative contributions of allochthonous and autochthonous organic matter and the potential effects these different food web pathways could have on pelagic and demersal fish consumers. Some estuarine fish species travel between many different habitats in a single day, making it challenging to estimate relative prey contributions from each habitat without information about movement. Stomach contents are frequently used to evaluate fish diet, but they can only provide a snapshot of consumption, whereas estuarine ecosystem processes operate at much broader spatiotemporal scales. Rapid rates of digestion and differences in the relative rates of deterioration between soft-bodied and hard-bodied prey can add further complication to fish stomach content analysis [41, 42]. Moreover, for primary consumers (i.e., invertebrates), stomach content analysis is inherently challenging, and the identification process can be both costly and time consuming.

Stable isotopes offer an alternative means to quantify food web pathways, and can be used either in lieu of or in tandem with stomach content analyses. In estuaries and other coastal ecosystems, ^13^C, ^15^N, and ^34^S isotopes have improved our ability to elucidate food web structure throughout a mosaic of interconnected habitat types with varying salinities and degrees of terrestrial influence [17, 20, 43–45]. Carbon isotope signatures differ among benthic, pelagic, and terrestrial primary producers as a result of the varying roles they play in the carbon cycle, while sulfur isotope signatures are heavily influenced by the reduction of sulfate in marine sediments along a salinity gradient [46]. Consequently, both are useful for identifying different sources of organic matter and their relative contributions to consumer diets in coastal ecosystems. Nitrogen isotope signatures, on the other hand, indicate the relative trophic position of consumers within the food web because they fractionate across trophic levels [47]. When used in tandem, these isotopes can strengthen our understanding of estuarine food web structure and processes—an important undertaking given widespread threats to coastal habitat from anthropogenic development and climate change.

Anthropogenic influence on the world’s coastal ecosystems has magnified the need to elucidate food web pathways. For example, it is estimated that 85% of historic tidal wetlands along the Pacific Coast of the United States have been lost to development, including about 95% of tidal wetlands in major river deltas [48]. Losses of vegetated riparian and emergent marsh habitats have resulted in reduced allochthonous inputs of organic matter, substantially disrupted energy flows, and reduced net primary production [49–51]. Efforts to restore estuarine landscapes, especially tidal wetlands, are often motivated by the need for high quality habitat and prey resources for threatened fish and wildlife; however, restoration success is largely dependent on a site’s connectivity with the surrounding habitat mosaic [15, 17, 52–54]. Integrated, estuary-wide assessments facilitated by stable isotope analysis may yield a more complete understanding of how emergent marshes contribute to food web structure and energy flows across interconnected habitats and trophic levels. This information is vital for informing restoration planning, habitat conservation, and other management decisions.

Estuarine restoration is seen as an important management tool to aid in the recovery of diadromous species, especially salmonids (*Oncorhynchus* spp.) listed under the Endangered Species Act (ESA; 64 FR 14308). Along the U.S. Pacific Coast, Pacific salmon use the estuarine habitat mosaic to acclimate to increasingly saline conditions before migrating to the open ocean [55]. Chinook salmon (*O*. *tshawytscha*) in particular can spend months at a time rearing in the estuary [56, 57]. Accessible and productive estuarine habitats that provide high-quality prey resources may enhance their growth and likelihood of survival [58–61]. In addition to migratory Chinook salmon, numerous resident fish species benefit from estuaries. For example, demersal species such as Pacific staghorn sculpin (*Leptocottus armatus*) reside in the estuary and nearshore zones year-round. Salmon and sculpin likely occupy distinct trophic niches despite using the same habitats [62]. Thus, the relative organic matter and prey contributions for these migratory pelagic and resident demersal fish species can serve as a robust indicator of estuarine habitat quality and food web functions.

In Puget Sound, Washington, USA, little is known about relative contributions of allochthonous and autochthonous organic matter to the structure of the estuarine food web. Furthermore, analyses to-date have had difficulty characterizing complex linkages across the entire estuarine habitat mosaic and among the full suite of food resources from terrestrial, pelagic, and benthic strata. The objectives of this study were to 1) Elucidate pelagic and demersal food web structure and 2) Determine the relative contributions of allochthonous and autochthonous organic matter using an estuary-wide approach. To satisfy these objectives, we used carbon, nitrogen, and sulfur stable isotope data from the full ecosystem mosaic including multiple trophic levels, environmental strata, and habitats. We examined primary producers and primary consumers contributing to the diets of juvenile Chinook salmon—a pelagic species—and resident sculpin—a demersal species—to provide a broader understanding of estuarine habitat function and connectivity. We conducted our analysis using data from the Nisqually River Delta in southern Puget Sound. The habitat complexity of the Nisqually River Delta and its recent restoration status make it an optimal site at which to study food web processes. Furthermore, the structure and composition of the food web is of great interest to habitat managers who are concerned with the preservation of estuary-dependent species, like the federally-threatened Nisqually fall Chinook salmon stock. We expected allochthonous subsidies from tidal marsh primary producers to be widespread, with a fading signal along a river-to-ocean gradient. Furthermore, we expected pelagic-feeding, migratory juvenile Chinook salmon to exhibit greater dietary variability and dependence on allochthonous organic matter than demersal sculpin. Findings from this study will offer valuable insight into estuarine organic matter contributions, and a connectivity-minded approach will inform management actions that can promote stable and resilient food webs.

## Materials and methods

### Study area

The Nisqually River Delta (NRD) is a large estuary in southern Puget Sound, Washington, USA (47°4′48″N, 122°42′20″W). It is located at the terminus of the Nisqually River—a 130 km-long glacial river that originates at the southwestern base of Mount Rainier. The regional climate is wet and temperate, and the Nisqually River watershed receives roughly 1 m of rainfall each year. A complex of dams, including 100 m-tall Alder Dam, controls hydrological flows and traps approximately 92% of the system’s fluvial sediments [63]. Consequently, the NRD is sediment starved relative to other, similarly-sized Puget Sound estuaries. River discharge ranges from 28 to 57 m^3^ s^-1^ with peak flows as high as 368 m^3^ s^-1^ during the rainy season. Salinity levels in the estuary vary spatiotemporally from 0–5 psu in the tidally influenced riverine areas to 5–30 psu in the marsh and nearshore intertidal zones. The NRD is macrotidal, with an average tidal range of 4 m, a mean tidal level of 1.34 m, a mean higher-high water level of 4.11 m, and a mean lower-low water level of -1.07 m [64, 65]. Thus, the amount of marine influence in the lower reaches of the river is likely considerable, despite substantial riverine flows and predominantly southwesterly winds.

**Fig 1:**
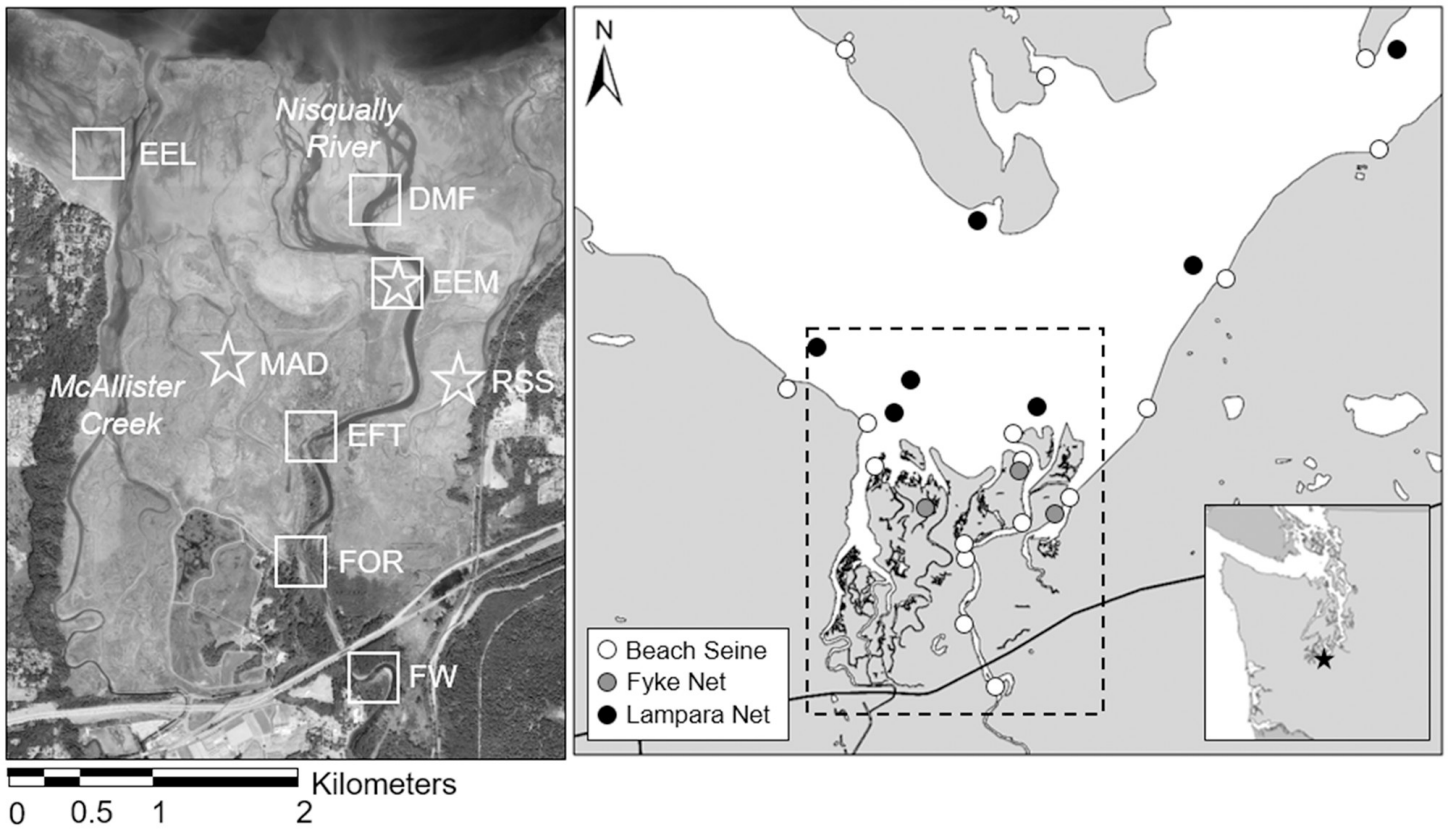
Map of stable isotope sampling sites in the Nisqually River Delta, Puget Sound, Washington, USA. Primary producers and invertebrate consumers were collected from the freshwater forested (FW), tidally influenced forested (FOR), transitional emergent marsh (EFT), estuarine emergent salt marsh (EEM), delta mudflat (DMF), and eelgrass (EEL) habitats of the Nisqually River Delta in 2015. We also sampled diatoms in the mudflats adjacent to historically-unaltered Animal Slough (EEM) and Red Salmon Slough (RSS) and in restored Madrone Slough (MAD) in 2011 (white stars). Circles in the right-hand panel represent locations where we captured juvenile Chinook salmon in 2011 and 2015 (all locations) and sculpin in 2011 (fyke nets only). Fish samples were collected across a broader spatial scale including the marine intertidal zone to account for the migratory behavior of juvenile salmon. Aerial imagery of the Nisqually River Delta (47°4′48″N, 122°42′20″W) was acquired by GeoTerra Inc. (Portland, Oregon, USA) in 2015.

The NRD is comprised of a diverse mosaic of riverine, marsh, and bare or vegetated (eelgrass, *Zostera marina*) intertidal habitat types [66–68] (Fig 1). Vegetative community structure and associated invertebrate communities vary spatially depending on salinity, inundation duration, and restoration status [18, 66, 69, 70]. Small scale tidal marsh restorations were implemented since 1996 and culminated in 2009 when several historical dikes were removed and over 308 ha were restored to tidal processes, resulting in variable conditions with respect to the influence of organic and inorganic sediments and the presence of salt marsh vegetation [19, 71, 72]. The historically unaltered salt marsh (including Animal and Red Salmon Sloughs) is fully vegetated with halophilic species such as saltgrass (*Distichlis spicata*), Lyngbye’s sedge (*Carex lyngbyei*), seaside arrowgrass (*Triglochin maritima*) and silverweed (*Potentilla anserina*) while the 2009 restoration area (including Madrone Slough) is largely unvegetated with sparse coverage of sandspurry (*Spergularia* spp.) and pickleweed (*Salicornia pacifica*). The brackish transitional marsh is characterized by overhanging vegetation such as broadleaf cattails (*Typha latifolia*) and sedges (*Eleocharis* spp.). Woody species such as bigleaf maple (*Acer macrophyllum*), willow (*Salix* spp.), and salmonberry (*Rubus spectabilis*) line the edges of the Nisqually River and its distributary channels in tidally influenced freshwater habitat.

### Data collection

#### Primary producers

We collected particulate organic matter (POM), microphytobenthos, macroalgae, vascular plants, and wrack from six estuarine habitat types in the NRD: freshwater forested, tidally influenced forested, transitional emergent marsh, estuarine emergent salt marsh, mudflat, and eelgrass (Fig 1). These samples represent primary producers forming the base of the food web across benthic, aquatic, and terrestrial environmental strata. We sampled POM in May and June 2015 using multiple 5-gallon carboys to collect eight replicate water samples per-habitat type on separate dates (Table 1). We filtered each carboy through a 100 μm sieve in the laboratory to remove zooplankton. We then strained the sample through a 20 μm sieve to collect POM. We rinsed the contents of the 20 μm sieve through a vacuum pump, capturing particles onto two separate, pre-combusted, 6 μm glass fiber filters. One filter was used for stable isotope analysis and the other was reserved for fluorometry. Stable isotope samples were stored in a plastic whirl-pak^®^ bag, covered in aluminum foil, and frozen for shipment to the Northern Arizona University Colorado Plateau Stable Isotope Laboratory (CPSIL). Fluorometry samples were stored similarly prior to analysis by the University of California Davis Tahoe Environmental Research Center (TERC). Chlorophyll *a* and pheophytin were extracted at TERC by submerging each filter in 25 ml of methanol overnight at 4°C. Fluorometric analysis of the diluted extract was then performed using a calibrated Turner Designs 10AU fluorometer (San Jose, California, USA).

**Table 1:** Primary source stable isotope signatures.

Primary Source	Dates Collected	N	δ^13^C (‰)	δ^15^N (‰)	δ^34^S (‰)
*Source 1*: *Riverine POM*		*24*	*-25*.*82 ± 0*.*87*	*3*.*07 ± 0*.*75*	*N/A*
FW POM	May 6–June 5, 2015	8	-25.60 ± 0.90	2.96 ± 0.85	N/A
FOR POM	May 6–June 5, 2015	8	-26.31 ± 0.67	2.67 ± 0.61	N/A
EFT POM	May 6–June 5, 2015	8	-25.54 ± 0.88	3.57 ± 0.54	N/A
*Source 2*: *Marsh POM*		*8*	*-24*.*75 ± 0*.*50*	*5*.*56 ± 1*.*50*	*N/A*
EEM POM	May 6–June 5, 2015	8	-24.75 ± 0.50	5.56 ± 1.50	N/A
*Source 3*: *Delta POM*		*16*	*-21*.*21 ± 1*.*42*	*6*.*47 ± 1*.*49*	*N/A*
DMF POM	May 6–June 5, 2015	8	-21.81 ± 1.38	6.53 ± 0.86	N/A
EEL POM	May 6–June 5, 2015	8	-20.61 ± 1.27	6.42 ± 2.00	N/A
*Source 4*: *Marsh Diatoms*		*14*	*-21*.*59 ± 1*.*65*	*4*.*86 ± 1*.*19*	*N/A*
EEM Diatoms	June 13–June 27, 2011	14	-21.59 ± 1.65	4.86 ± 1.19	N/A
*Source 5*: *Delta Diatoms*		*24*	*-15*.*81 ± 0*.*71*	*1*.*98 ± 1*.*01*	*N/A*
MAD Diatoms	June 13–June 27, 2011	12	-15.53 ± 0.48	2.26 ± 0.86	N/A
RSS Diatoms	June 13–June 27, 2011	12	-16.10 ± 0.81	1.70 ± 1.11	N/A
*Source 6*: *Riverine Plants*		*72*	*-30*.*57 ± 1*.*66*	*-1*.*51 ± 1*.*39*	*7*.*95 ± 1*.*06*
FW *Rubus spectabilis*	May 6–May 26, 2015	12	-31.42 ± 1.36	-2.00 ± 1.03	7.34 ± 1.03
FW *Salix* sp.	May 6–May 26, 2015	6	-28.44 ± 0.87	-0.20 ± 0.71	7.80 ± 0.74
FW *Urtica dioica*	May 6–May 26, 2015	12	-31.61 ± 1.49	0.13 ± 1.11	8.33 ± 1.10
FW leaf litter	May 6–May 26, 2015	6	-28.53 ± 0.85	-0.81 ± 0.34	8.19 ± 1.39
FOR *Acer macrophyllum*	May 6–May 26, 2015	6	-30.15 ± 0.71	-2.76 ± 0.77	8.22 ± 0.84
FOR *Rubus spectabilis*	April 22–May 26, 2015	12	-31.53 ± 1.44	-1.69 ± 1.40	8.31 ± 1.33
FOR *Urtica dioica*	April 22–May 26, 2015	12	-30.38 ± 1.48	-2.35 ± 0.93	7.83 ± 0.76
FOR leaf litter	May 6–May 28, 2015	6	-29.86 ± 0.87	-2.51 ± 0.63	7.61 ± 0.92
*Source 7*: *Marsh C3 Plants*		*30*	*-27*.*34 ± 1*.*21*	*4*.*79 ± 1*.*42*	*12*.*00 ± 5*.*89*
EFT *Carex lyngbyei*	May 6–May 26, 2015	6	-28.34 ± 0.96	4.24 ± 1.62	12.90 ± 5.54
EFT *Eleocharis acicularis*	May 6–May 26, 2015	6	-28.46 ± 0.61	4.47 ± 1.34	15.66 ± 3.34
EFT *Typha latifolia*	May 6–May 26, 2015	6	-27.03 ± 0.51	5.49 ± 1.27	11.45 ± 3.31
EEM *Carex lyngbyei*	May 6–May 26, 2015	6	-25.70 ± 0.64	4.90 ± 1.13	2.10 ± 3.61
EEM *Salicornia pacifica*	May 6–May 26, 2015	6	-27.16 ± 1.08	3.47 ± 1.29	16.04 ± 1.46
*Source 8*: *Marsh C4 Plants*		*12*	*-15*.*46 ± 1*.*86*	*4*.*76 ± 0*.*80*	*3*.*77 ± 4*.*1*
EEM *Distichlis spicata*	April 22–May 26, 2015	12	-15.46 ± 1.86	4.76 ± 0.80	3.77 ± 4.1
*Source 9*: *Delta Plants*		*6*	*-10*.*95 ± 1*.*85*	*7*.*94 ± 1*.*25*	*19*.*81 ± 3*.*92*
DMF *Ulva* spp	May 8–May 27, 2015	6	-12.02 ± 1.84	7.91 ± 0.96	22.53 ± 1.04
EEL *Ulva* spp	May 6–May 27, 2015	5	-10.56 ± 2.45	8.77 ± 1.70	22.33 ± 1.53
EEL *Zostera marina*	May 6–May 28, 2015	6	-10.36 ± 0.59	7.28 ± 0.77	14.99 ± 1.72

Stable isotope signatures (mean ± SD), collection dates, and number of samples collected (*N*) for primary sources in the Nisqually River Delta [84]. A hierarchical clustering analysis determined broader source groupings by primary producer and habitat type, with mean ± SD isotopic signatures for each source indicated in italics. Sources were used in a Bayesian stable isotope mixing model to evaluate relative contributions of primary producers to invertebrate consumer diets. Vascular plant groups include above- and belowground biomass and wrack. Habitat abbreviations are as follows: freshwater forested (FW), tidally influenced forested (FOR), transitional emergent marsh (EFT), estuarine emergent salt marsh (EEM), delta mudflat (DMF), eelgrass (EEL), Madrone Slough (MAD; a restoring emergent marsh), and Red Salmon Slough (RSS; a relict emergent marsh).

We sampled microphytobenthos (hereafter, diatoms) by using a spatula or toothbrush to scrape biofilm from the top 1–2 mm of the exposed marsh surface. We collected three replicate samples of roughly 40 g each at each site, with replicates spaced more than 50 m apart. We stored biofilm in plastic bags covered in aluminum foil and kept them on ice while in the field. Samples were shipped overnight to the USGS San Francisco Bay Field Station Invertebrate Ecology Laboratory (IEL). To extract diatoms, sediment was spread to 1 cm depth on a shallow pan and covered with a 63 μm mesh nylon screen with glass wool fibers placed on top. The glass wool was sprayed with a filtered (0.2 μm mesh) sea salt solution and left for 24 hours under laboratory lights to allow for the vertical movement of the diatoms onto the fibers [73]. These fibers were then frozen and shipped to CPSIL for stable isotope analysis. We were unable to successfully extract sufficient amounts of diatoms from the samples collected in transitional, emergent salt marsh, and mudflat habitats in 2015, so as a proxy we used data from samples that had been collected from the estuarine emergent salt marsh site (Animal Slough), historically unaltered Red Salmon Slough, and restored Madrone Slough in June 2011. Isotopic signatures for other primary producers at the emergent salt marsh site (POM, macroalgae, Lyngbye’s sedge, and saltgrass) were nearly identical in 2011 and 2015 (all within ± 1 SD of sample variation), indicating that among-year differences in stable isotope signatures for the base of the food web were likely minimal.

We collected brown algae (*Fucus spp*) from the emergent salt marsh and sea lettuce (*Ulva spp*) from the mudflat and eelgrass habitats in May 2015. We collected six replicate samples per-site spaced at least 50 m apart. Each sample was stored in a plastic bag and kept on ice in the field. After returning to the laboratory, we immediately triple washed each sample to remove invertebrates and debris. Samples were clipped to 30 g wet biomass and refrigerated at 5°C prior to shipment to CPSIL.

We sampled above- and belowground biomass and detritus from vascular plants in the freshwater, tidal-forested, transitional, emergent salt marsh, and eelgrass sites in April and May 2015. We collected three replicate samples of representative plant species at each site, and aboveground, belowground, and detrital samples were processed separately. In the freshwater and tidal-forested habitats, we sampled salmonberry (*Rubus spectabilis*), willow, maple, and stinging nettle (*Urtica dioica*). Detritus was comprised of leaf litter from multiple plant species. In the transitional marsh we sampled broad-leaf cattail, needle spikerush (*Eleocharis acicularis*), and Lyngbye’s sedge. In the emergent salt marsh, we sampled Lyngbye’s sedge, pickleweed, and saltgrass (the only primary producer at our study sites that derives its energy from the C4 photosynthetic pathway). Detritus at the transitional and emergent salt marsh sites was comprised primarily of dead vascular plant material deposited by tides, or wrack. In the eelgrass habitat, we collected eelgrass shoots by pulling an individual stalk from the root. We triple washed each sample with deionized water to remove epifauna and debris. Samples of 10 g wet plant matter were stored in plastic bags at 5°C prior to shipment to CPSIL.

#### Invertebrate consumers

Invertebrates are important components of the estuarine food web and serve as primary sources of prey for salmon, sculpin, and other species of fish. We sampled benthic, aquatic, and terrestrial invertebrate consumers (if present) in all six habitat types from May–July 2015 (Table 2). Collection efforts were distributed throughout each site to account for spatial heterogeneity in invertebrate taxa. We collected invertebrates from the epibenthos using a 10 cm diameter clam gun at low tide to extract stratified sediment cores. Each core was rinsed through a 0.5 mm sieve using filtered seawater to separate invertebrates from the substrate. The remaining invertebrates were then gently transferred to a 250 ml jar using tweezers and stored on ice in the field. To collect aquatic invertebrates from the water column, we towed a 50 cm diameter, 150 cm long, 153 μm mesh neuston net from the edge of a boat for up to 10 minutes. The cod end was then rinsed into a 250 ml jar using filtered seawater. At the eelgrass site, we collected aquatic epifaunal samples by picking eelgrass shoots from the root by hand and transferring them to a 250 ml jar of filtered seawater. Epifauna were later gently rinsed or tweezed from eelgrass shoots in the lab. Terrestrial invertebrates were collected from the freshwater, tidal-forested, transitional, and emergent salt marsh habitats. We used a muslin sweep net to sample from vegetation along the river or channel edge. We transferred live invertebrates to an inflated plastic bag while in the field to prevent crushing. Upon return to the laboratory, all invertebrate samples were stored at 5°C in a refrigerator prior to sorting or shipment.

**Table 2:** Invertebrate and fish stable isotope signatures.

Consumer	Dates Collected	N	δ^13^C (‰)	δ^15^N (‰)	δ^34^S (‰)
*Terr 1*: *Riverine Insects*		*8*	*-26*.*25 ± 2*.*08*	*1*.*77 ± 2*.*23*	*6*.*58 ± 1*.*44*
Coleoptera–Coccinellidae	June 8–June 12, 2015	1	-25.29 ± N/A	2.58 ± N/A	7.57 ± N/A
Coleoptera–Coleoptera (other)	June 8–June 12, 2015	1	-23.47 ± N/A	2.63 ± N/A	7.58 ± N/A
Hemiptera–Aphidae	June 8–June 12, 2015	2	-25.93 ± 2.85	0.59 ± 2.87	5.89 ± 2.07
Hemiptera–Cicadellidae	June 8–June 12, 2015	1	-25.31 ± N/A	4.53 ± N/A	6.31 ± N/A
Hymenoptera	June 8–June 12, 2015	2	-27.38 ± 0.83	2.29 ± 2.09	6.52 ± 2.68
Lepidoptera	June 8–June 12, 2015	1	-29.28 ± N/A	-1.37 ± N/A	6.38 ± N/A
*Terr 2*: *Marsh Insects*		*5*	*-24*.*01 ± 2*.*14*	*5*.*77 ± 1*.*31*	*8*.*76 ± 5*.*65*
Hemiptera–Cicadellidae	June 8–June 12, 2015	3	-23.12 ± 2.33	4.90 ± 0.74	6.80 ± 6.36
Hemiptera–Lygaeidae	June 8–July 3, 2015	1	-24.48 ± N/A	7.07 ± N/A	14.69 ± N/A
Lepidoptera	June 8–June 12, 2015	1	-26.22 ± N/A	7.09 ± N/A	8.72 ± N/A
*Terr 3*: *Saldidae*		*3*	*-19*.*40 ± 0*.*69*	*7*.*87 ± 0*.*73*	*9*.*16 ± 4*.*92*
Hemiptera–Saldidae	June 8–July 3, 2015	3	-19.40 ± 0.69	7.87 ± 0.73	9.16 ± 4.92
*Terr 4*: *Dolichopodidae*		*12*	*-21*.*41 ± 1*.*21*	*8*.*98 ± 1*.*07*	*9*.*81 ± 2*.*92*
Diptera–Dolichopodidae	June 8–July 3, 2015	12	-21.41 ± 1.21	8.98 ± 1.07	9.81 ± 2.92
*Terr 5*: *Ephydridae*		*11*	*-23*.*77 ± 2*.*38*	*8*.*95 ± 1*.*07*	*10*.*16 ± 2*.*01*
Diptera–Ephydridae	June 8–July 3, 2015	11	-23.77 ± 2.38	8.95 ± 1.07	10.16 ± 2.01
*Terr 6*: *Riverine Dipterans*		*7*	*-30*.*10 ± 4*.*37*	*6*.*30 ± 1*.*47*	*9*.*23 ± 1*.*36*
Diptera–Brachycera	June 8–July 3, 2015	2	-25.70 ± 1.17	6.35 ± 0.26	8.41 ± 0.93
Diptera–Chironomidae	June 8–June 12, 2015	4	-33.26 ± 2.59	7.01 ± 0.88	10.27 ± 0.88
Diptera–Sciomyzidae	June 8–June 12, 2015	1	-26.29 ± N/A	3.38 ± N/A	7.75 ± N/A
*Terr 7*: *Marsh Dipterans*		*4*	*-22*.*31 ± 3*.*24*	*7*.*28 ± 0*.*20*	*10*.*92 ± 2*.*77*
Diptera–Chironomidae	June 8–June 12, 2015	1	-25.74 ± N/A	7.57 ± N/A	7.92 ± N/A
Diptera–Muscidae	June 8–June 12, 2015	1	-24.31 ± N/A	7.14 ± N/A	9.21 ± N/A
Diptera–Tipulidae	June 8–June 12, 2015	2	-19.60 ± 1.04	7.21 ± 0.06	13.27 ± 0.37
*Aq 1*: *Riverine Isopods*		*3*	*-19*.*59 ± 2*.*35*	*7*.*60 ± 0*.*45*	*19*.*47 ± 0*.*09*
*Gnorimosphaeroma oregonensis*	May 28–June 3, 2015	3	-19.59 ± 2.35	7.60 ± 0.45	19.47 ± 0.09
*Aq 2*: *Marsh Isopods*		*3*	*-17*.*24 ± 0*.*77*	*8*.*07 ± 0*.*36*	*20*.*08 ± 1*.*05*
*Gnorimosphaeroma oregonensis*	June 2–June 3, 2015	3	-17.24 ± 0.77	8.07 ± 0.36	20.08 ± 1.05
*Aq 3*: *Riverine Mysids*		*11*	*-17*.*33 ± 4*.*94*	*10*.*57 ± 3*.*26*	*16*.*21 ± 0*.*51*
*Neomysis mercedis*	June 2–June 3, 2015	11	-17.33 ± 4.94	10.57 ± 3.26	16.21 ± 0.51
*Aq 4*: *Marsh Mysids*		*3*	*-14*.*59 ± 0*.*16*	*11*.*29 ± 0*.*44*	*17*.*72 ± 0*.*17*
*Neomysis mercedis*	June 2–June 3, 2015	3	-14.59 ± 0.16	11.29 ± 0.44	17.72 ± 0.17
*Aq 5*: *Delta Mysids*		*8*	*-13*.*99 ± 1*.*72*	*10*.*98 ± 0*.*81*	*17*.*05 ± 0*.*59*
*Neomysis mercedis*	June 2–June 3, 2015	8	-13.99 ± 1.72	10.98 ± 0.81	17.05 ± 0.59
*Aq 6*: *Delta Shrimp*		*3*	*-13*.*23 ± 0*.*26*	*12 ± 0*.*16*	*18*.*02 ± 0*.*55*
*Crangon* sp.	June 2–June 3, 2015	3	-13.23 ± 0.26	12 ± 0.16	18.02 ± 0.55
*Ben 1*: *Riverine Crustaceans*		*11*	*-20*.*79 ± 2*.*83*	*8*.*75 ± 2*.*28*	*15*.*03 ± 3*.*04*
Amphipoda–*Ampithoe* sp.	June 2–June 5, 2015	4	-21.88 ± 3.33	7.61 ± 1.63	16.17 ± 3.96
Amphipoda–Corophiidae	June 2–June 8, 2015	6	-20.37 ± 2.74	9.80 ± 2.40	14.07 ± 2.57
Cumacea	June 2–June 5, 2015	1	-18.96 ± N/A	6.96 ± N/A	16.25 ± N/A
*Ben 2*: *Marsh Crustaceans*		*12*	*-17*.*20 ± 1*.*28*	*10*.*28 ± 1*.*44*	*15*.*80 ± 2*.*43*
Amphipoda–*Ampithoe* sp.	June 2–June 5, 2015	5	-17.87 ± 1.78	9.78 ± 1.17	15.99 ± 3.04
Amphipoda–Corophiidae	June 2–June 8, 2015	6	-16.54 ± 0.19	10.88 ± 1.60	15.68 ± 2.26
Copepoda–Harpacticoida	June 2–June 5, 2015	1	-17.74 ± N/A	9.25 ± N/A	N/A
*Ben 3*: *Delta Crustaceans*		*16*	*-13*.*53 ± 1*.*74*	*9*.*73 ± 1*.*01*	*16*.*11 ± 1*.*61*
Amphipoda–*Ampithoe* sp.	June 2–June 5, 2015	5	-14.50 ± 1.55	9.39 ± 0.39	16.99 ± 1.29
Amphipoda–Corophiidae	May 28–June 8, 2015	6	-14.24 ± 1.14	9.82 ± 0.13	16.07 ± 1.50
Amphipoda–*Eogammarus* sp.	June 2–June 5, 2015	1	-11.63 ± N/A	12.90 ± N/A	16.54 ± N/A
Copepoda–Harpacticoida	June 2–June 5, 2015	1	-11.74 ± N/A	9.63 ± N/A	N/A
Cumacea	June 2–June 5, 2015	2	-10.87 ± 0.76	8.46 ± 0.59	13.11 ± N/A
Tanaidacea	June 2–June 5, 2015	1	-13.44 ± N/A	10.35 ± N/A	14.50 ± N/A
*Ben 4*: *Riverine Larvae*		*12*	*-21*.*10 ± 3*.*96*	*7*.*05 ± 1*.*16*	*12*.*18 ± 1*.*33*
Diptera–Ceraptogonidae	June 2–June 5, 2015	2	-18.14 ± 3.55	6.55 ± 0.95	N/A
Diptera–Chironomidae	June 2–July 3, 2015	5	-21.33 ± 1.36	7.69 ± 1.48	11.49 ± 1.49
Diptera–Dolichopodidae	June 2–June 5, 2015	1	-16.89 ± N/A	7.71 ± N/A	14.07 ± N/A
Diptera–Trichoptera	June 2–June 5, 2015	4	-23.34 ± 5.69	6.35 ± 0.19	12.57 ± 0.43
*Ben 5*: *Riverine Polychaetes*		*8*	*-23*.*43 ± 3*.*80*	*9*.*12 ± 1*.*74*	*12*.*13 ± 4*.*29*
*Neanthes* sp.	May 28–June 8, 2015	8	-23.43 ± 3.80	9.12 ± 1.74	12.13 ± 4.29
*Ben 6*: *Delta Polychaetes*		*20*	*-14*.*01 ± 1*.*77*	*13*.*32 ± 2*.*92*	*15*.*73 ± 30*
*Eteone* sp.	June 2–June 8, 2015	6	-12.86 ± 0.56	15.26 ± 0.91	15.64 ± 0.14
*Glycera nana*	June 2–June 8, 2015	2	-13.89 ± 0.02	14.98 ± 0.46	15.39 ± 0.67
*Goniada brunea*	June 2–June 8, 2015	2	-15.22 ± 0.17	13.04 ± 0.61	10.96 ± 2.35
*Neanthes* sp.	May 28–June 8, 2015	3	-13.14 ± 0.12	12.95 ± 1.46	14.85 ± 3.44
Phyllodocidae	June 2–June 8, 2015	1	-12.78 ± N/A	14.52 ± N/A	15.32 ± N/A
Sabellidae	June 2–June 8, 2015	1	-13.62 ± N/A	14.92 ± N/A	17.39 ± N/A
Spionidae	June 2–June 8, 2015	5	-15.79 ± 2.59	10.11 ± 4.17	19.37 ± 0.34
*Fish 1*: *Riverine Salmon (2015)*	*May 27*, *2015*	*4*	*-20*.*49 ± 1*.*25*	*11*.*10 ± 1*.*11*	*11*.*39 ± 5*.*22*
*Fish 2*: *Marsh Salmon (2015)*	*May 22*–*July 7*, *2015*	*19*	*-19*.*26 ± 2*.*19*	*11*.*56 ± 1*.*30*	*12*.*61 ± 3*.*84*
*Fish 3*: *Delta Salmon (2015)*	*May 22*–*July 7*, *2015*	*8*	*-18*.*27 ± 2*.*57*	*11*.*15 ± 1*.*34*	*11*.*97 ± 3*.*11*
*Fish 4*: *Riverine Salmon (2011)*	*May 11*–*May 25*, *2011*	*10*	*-24*.*84 ± 1*.*74*	*10*.*68 ± 0*.*67*	*7*.*29 ± 1*.*65*
*Fish 5*: *Marsh Salmon (2011)*	*June 6*–*June 23*, *2011*	*10*	*-21*.*03 ± 3*.*97*	*11*.*59 ± 1*.*10*	*11*.*04 ± 3*.*29*
*Fish 6*: *Delta Salmon (2011)*	*June 8*–*June 24*, *2011*	*14*	*-18*.*93 ± 2*.*42*	*11*.*35 ± 1*.*54*	*12*.*65 ± 2*.*98*
*Fish 7*: *Marsh Sculpin (2011)*	*June 6*–*June 23*, *2011*	*15*	*-15*.*98 ± 1*.*50*	*12*.*44 ± 0*.*43*	*15*.*46 ± 1*.*29*

Stable isotope signatures (mean ± SD), collection dates, and number of composite samples collected (*N*) for terrestrial (Terr), aquatic (Aq), and benthic (Ben) invertebrates and fish in the Nisqually River Delta [84]. Isotopic signatures for broader consumer groups are shown in italics. The “riverine” groups include invertebrates and fish from the freshwater and tidal-forested habitats, the “marsh” groups from the transitional and emergent salt marsh habitats, and the “delta” groups from the mudflat and eelgrass habitats unless otherwise specified. Consumer groups were used in a Bayesian stable isotope mixing model to evaluate relative contributions of primary producers to invertebrate consumer diets and relative contributions of invertebrates to fish consumer diets.

We shipped benthic and aquatic invertebrates to IEL within three days of collection, while terrestrial insects were processed on-site within 24-hours of collection. All invertebrate samples were refrigerated overnight to allow for gut evacuation. We used tweezers or forceps to separate live invertebrates from other debris before grouping and weighing by taxon. Invertebrates were initially grouped into 39 taxonomic categories ranging from family to species using a stereo-dissection microscope at 7–45× magnification. We collected three replicate samples per taxon whenever possible, but some very small or uncommon invertebrate consumers were later grouped together by habitat, environmental stratum, and feeding guild to accumulate the necessary biomass for stable isotope processing. All invertebrates were triple washed with deionized water to rinse off debris and tools were washed with 5% HCl solution followed by a deionized water rinse between handling each sample to prevent cross-contamination. Samples were frozen in -20°C prior to shipping to CPSIL for isotope analyses.

#### Fish consumers

We collected and analyzed a pelagic fish species (juvenile Chinook salmon) and a demersal fish species (Pacific staghorn sculpin) to represent the upper trophic levels of the NRD aquatic food web. During the juvenile Chinook salmon outmigration period (May–July) of 2011 and 2015, we collected juvenile Chinook salmon without adipose fin clips (presumed wild-origin) from representative sites within the estuarine habitat mosaic (Fig 1; Table 2). We used a fyke net to catch fish in tidal sloughs that dewatered with the tide, a beach seine to catch fish along the shoreline in the river and estuary, and a lampara net to catch juvenile salmon in the marine intertidal zone. Up to 10 juvenile Chinook salmon were euthanized for study purposes per-site, including 41 unmarked fish in 2011 and 31 unmarked fish in 2015 that were used for stable isotope analysis. We also collected 21 sculpin from fyke nets set in emergent salt marsh in 2011. All fish were caught, handled, and euthanized (with Tricaine methano-sulfate and placed on ice) under the auspices of a Puget Sound Tribal Salmon Research Plan (in accordance to the ESA 4(d) rule for Tribal Plans (50 CFR 223.204; 65 FR 42481]) coordinated by the Northwest Indian Fisheries Commission. Hatchery-reared (marked) Chinook salmon were omitted from the analysis because their diets were more likely to be comprised of hatchery food in the weeks prior to capture [68]. All samples were frozen at -20°C in a laboratory freezer. Capture and processing procedures are described in detail in Ellings et al. [72] and Davis et al. [61, 68], and described briefly below.

To prepare fish tissues for stable isotope analysis, we used a scalpel cleaned with 5% HCl solution and rinsed with deionized water to extract muscle tissue from above the lateral line on the flank end of the fish. The muscle tissue was separated from bone, skin, and scales and rinsed with deionized water. Tissue samples were frozen in plastic bags at -20°C and shipped on dry ice to CPSIL for analysis.

### Stable isotope measurements

We shipped primary producer and consumer tissue samples on dry ice to CPSIL for carbon (^13^C), nitrogen (^15^N), and sulfur isotope (^34^S) analysis. Each sample was dried in a laboratory oven for 24 hours at 80°C and pulverized using a ball mill grinder or mortar and pestle. The dried tissue was then weighed on a 0.01 mg accuracy scale (0.6–1.2 mg for ^13^C and ^15^N and 4–6 mg for ^34^S) and packed into a 4 × 6 mm tin capsule. Carbon and nitrogen samples were run on a Thermo-Electron Delta V Advantage (Thermo Scientific, Waltham, Massachusetts, USA) isotope ratio mass spectrometer (IRMS) configured through a Finnigan ConFlo III (Thermo-Finnigan, Breman, Germany) and interfaced with a Carlo Erba NC2100 elemental analyzer (Carlo Erba Instruments, Milan, Italy) for combustion and separation of ^13^C and ^15^N. For ^34^S, a DeltaPlus Advantage IRMS (Thermo Fisher Scientific, Bremen, Germany) was configured through a Finnigan ConFlo III and interfaced with a Costech ECS4010 elemental analyzer (Costech Analytical, Valencie, CA, USA). Peach leaves (NIST 1547) were used as the internal laboratory working standard for ^13^C and ^15^N and bovine liver (NIST 1577) was used for ^34^S. Standards were interspersed throughout the run roughly every 10 samples to check for drift and were included at the end of each run as a weight series to check for linearity. More information is available at http://www.isotope.nau.edu.

Stable isotopes of carbon, nitrogen, and sulfur were expressed in standard delta notation:

$$\delta^{H}X=\left(\frac{R_{sample}}{R_{standard}}-1\right)\times 1,000$$ (1)

where *X* = δ^13^C, δ^15^N, or δ^34^S and *R* is the ratio of heavy and light isotopes in a sample. We expressed stable isotope ratios as per mil (‰, or parts per thousand) differences relative to the international standards Pee Dee belemnite for δ^13^C, atmospheric nitrogen for δ^15^N, and Vienna-Canyon Diablo troilite for δ^34^S. The δ^13^C values of invertebrate and fish samples with a C:N ratio more than 3.5 were manually corrected for lipid depletion according to the equations found in Post et al. [47].

We calculated each consumer’s trophic level (TL) using the formula from Vander Zanden and Rasmussen [74]:

$$TL=\;\frac{\delta^{15}N_{Consumer}-\delta^{15}N_{Baseline}}{TEF}+\lambda$$ (2)

where δ^15^N_Consumer_ is the δ^15^N value of the consumer or predator in question and δ^15^N_Baseline_ is the average δ^15^N of the consumers at the base of the food web (in this case, herbivorous terrestrial insects; λ = 2). We used a trophic enrichment factor (TEF) based on Davis et al.’s [68] estimate of 2.91 ± 0.38‰ for juvenile Chinook salmon.

### Statistical analysis

We used multivariate analyses and a Bayesian stable isotope mixing model (SIMM) [75] to evaluate among-group differences in invertebrate and fish consumer diets and to elucidate food web pathways for the NRD. We used a hierarchical clustering analysis with Ward’s minimum variance method to sort primary producers into distinct, biologically relevant source groups for the SIMM [76, 77]. The clustering procedure was applied to a Euclidean distance matrix of δ^13^C, δ^15^N, and δ^34^S signatures, where POM, diatoms, macroalgae, and vascular plants were treated separately. Primary producers were clustered by broader habitat type (i.e., riverine, marsh, delta) for POM, diatoms, and macroalgae, and by habitat type and taxon for vascular plants (Table 1; see [19]).

For POM, we analyzed chlorophyll *a* and pheophytin to estimate the relative levels of autochthonous phytoplankton (as opposed to allochthonous detritus) for each primary source group. Chlorophyll *a* was considered an indicator of live phytoplankton, while pheophytin was used as indicator of dead phytoplankton (e.g., as a result of freshwater exposure). We used a generalized linear model with a log-link gaussian function to analyze differences in the concentration of each compound by habitat type. When appropriate, we used Tukey’s Honestly Significant Difference (Tukey HSD) to identify habitats that had comparatively higher or lower concentrations of chlorophyll *a* and pheophytin. We also examined mean C:N ratios and δ^13^C values to determine POM composition. Phytoplankton-dominant POM tends to have δ^13^C values between -22 and -20‰ and a C:N ratio between 5 and 9, whereas POM derived from terrestrial plant detritus tends to have a C:N ratio between 15 and 50 [78–80].

For invertebrate consumers, we used the hierarchical clustering procedure described above to sort taxa into distinct, biologically relevant groups for analysis. Clusters were further grouped based on environmental strata, habitat type, and taxonomy (Table 2). We used a permutational multivariate analysis of variance (PERMANOVA) with 1,000 permutations on a Euclidean distance matrix of δ^13^C, δ^15^N, and δ^34^S to identify differences in the stable isotope signatures of invertebrate consumers. We evaluated each environmental stratum separately and used the Pillai’s trace test statistic to identify significant pairwise differences among taxonomic groups within strata. Output from this analysis was used to inform broader invertebrate source groupings for the SIMM examining the diets of pelagic and demersal fish. All multivariate analyses were conducted using the “vegan” package in R 3.5.1 [81].

We used two separate SIMMs to derive posterior estimates of food web linkages among primary producers, invertebrate consumers, and fish consumers in different habitat types; the “invertebrate consumer” SIMM identified primary producers with the greatest likelihood of contributing to invertebrate diets and the “fish consumer” SIMM identified invertebrate prey with the greatest likelihood of contributing to pelagic and demersal fish diets. For fish, we also calculated the proportional contribution of allochthonous (riverine, C3, and C4 marsh plants) versus autochthonous organic matter (POM, diatoms, and algae) to diets based on output from both SIMMs. Sulfur was omitted from the invertebrate consumer SIMM because we did not collect enough sample biomass to measure δ^34^S for POM or diatoms. We conducted analyses using the “IsotopeR” package in R [82], which allows for the inclusion of measurement error and trophic enrichment with discrimination error. We used a measurement error of 0.04‰ for δ^13^C, 0.07‰ for δ^15^N, and 0.12‰ for δ^34^S based on the precision determined for the peach leaf and bovine liver standards used by CPSIL. For the invertebrate consumer SIMM, we used a TEF of δ^13^C = 0.42 ± 0.18‰ and δ^15^N = 2.22 ± 0.28‰ based on literature values for aquatic invertebrates [83]. For the fish consumer SIMM, we used the TEF values of δ^13^C = -0.21 ± 0.20‰, δ^15^N = 2.91 ± 0.38‰, and δ^34^S = -0.20 ± 0.29‰ from Davis et al. [68]. Both SIMMs were run using an uninformative prior based on the Dirichlet distribution, and each model was set to run three chains for 300,000 Markov Chain Monte Carlo simulations with a burn-in of 150,000.

## Results

### Isotopic separation of primary sources

The hierarchical clustering analysis of δ^13^C, δ^15^N, and δ^34^S (when available) sorted primary sources into distinct, biologically relevant groups. For POM, δ^13^C and δ^15^N signatures indicated that samples were best split into three groups based on habitat type. The first group, “riverine POM,” was the most depleted in δ^13^C and δ^15^N, and consisted of POM samples from the freshwater, tidal-forested, and transitional habitats (Table 1). The second group, “marsh POM,” represented intermediate δ^13^C and δ^15^N values and consisted of samples collected only from the emergent salt marsh habitat. The third group, “delta POM,” was comprised of samples from the mudflat and eelgrass habitats, which had the most enriched δ^13^C and δ^15^N signatures.

These groupings were partially representative of among-habitat differences in chlorophyll *a* concentrations, which we used as a proxy for live phytoplankton presence. Chlorophyll *a* was significantly higher in freshwater habitat and significantly lower in emergent salt marsh than in any of the other four habitat types (*F*_*5*,*30*_ = 3.15, *P* = 0.021; Means ± SD: freshwater = 19.68 ± 13.94 μg/L, tidal-forested = 12.85 ± 3.88 μg/L, transitional = 9.08 ± 4.40 μg/L, emergent salt marsh = 5.66 ± 4.18 μg/L, mudflat = 12.84 ± 5.09 μg/L, eelgrass = 7.61 ± 3.83 μg/L). Pheophytin, an indicator of dead phytoplankton, was also highest in freshwater and lowest in emergent salt marsh, but this effect was marginally significant (*F*_*5*,*30*_ = 2.34, *P* = 0.066; Means ± SD: freshwater = 15.84 ± 9.79 μg/L, tidal-forested = 10.39 ± 3.40 μg/L, transitional = 8.91 ± 4.42 μg/L, emergent salt marsh = 5.00 ± 4.28 μg/L, mudflat = 11.44 ± 10.44 μg/L, eelgrass = 5.03 ± 2.85 μg/L). These results would suggest that phytoplankton was most prevalent in the riverine POM primary source group, followed by the delta POM and marsh POM groups; however, C:N ratios were slightly > 9 in the freshwater (11.23), tidal-forested (10.75), transitional (11.48), and estuarine (9.65) habitats. As such, riverine POM likely comprised of a mix of autochthonous phytoplankton and allochthonous terrestrial detritus, while delta POM (mudflat C:N = 7.17, eelgrass C:N = 6.12) was phytoplankton-dominant.

For diatoms, δ^13^C and δ^15^N signatures pointed to two distinct source groupings. “Marsh diatoms” represented samples collected from the edges of tidal sloughs running through emergent salt marsh (Animal Slough). The δ^13^C values of marsh diatoms were similar to that of delta POM, although δ^15^N was moderately lower (Table 1). The second source group of diatoms, which we termed “delta diatoms,” consisted of samples from Madrone Slough and Red Salmon Slough. Although spatially further apart from each other than from the emergent salt marsh site, δ^13^C and δ^15^N signatures of diatoms from these two sites were highly similar. δ^13^C values were about 6‰ more enriched for delta diatoms than for marsh diatoms, while δ^15^N values were about 3‰ more depleted.

We opportunistically collected *Fucus* macroalgae in emergent salt marsh habitat and *Ulva* macroalgae in the mudflat and eelgrass habitats; however, the δ^13^C and δ^15^N signatures for *Fucus* (δ^13^C = -21.38 ± 0.21‰, δ^15^N = 5.89 ± 0.28‰) were indistinguishable from that of delta POM despite being spatially and biologically distinct, so we omitted it as a primary source. *Ulva* was the most highly enriched primary source for δ^15^N and δ^34^S, and the second most highly enriched source for δ^13^C. The clustering analysis indicated that macroalgal samples from the mudflat and eelgrass sites were highly similar.

For vascular plants, a clustering analysis using δ^13^C, δ^15^N, and δ^34^S identified five distinct groups. These primary source groups included above- and belowground biomass from the freshwater and tidal-forested habitats (“riverine plants”), aboveground biomass from C3 plants in the transitional and emergent salt marsh habitats (“marsh C3 plants”), belowground biomass from C3 plants in the transitional and emergent salt marsh habitats, above- and belowground biomass from the C4 plant *Distichlis spicata* in the emergent salt marsh habitat (“marsh C4 plants”), and above- and belowground biomass from *Zostera marina* shoots. Although we included belowground biomass in some primary source groups, aboveground biomass is more likely to be consumed by insects or enter the food web via detrital pathways. The stable isotope signatures for marsh belowground biomass (δ^13^C = -25.28 ± 3.84‰, δ^15^N = 4.88 ± 1.85‰, δ^34^S = 8.10 ± 6.66‰) were similar to the marsh C3 plant group and were also highly variable, so we had both a quantitative and ecological justification to omit it as a primary source in the SIMM. Likewise, after running an initial SIMM, we determined that the model could not differentiate between the algae *Ulva* and submerged aquatic eelgrass as primary sources using only δ^13^C and δ^15^N values, so we combined these into a single source which we called “delta plants.”

### Consumer trophic levels

**Fig 2:**
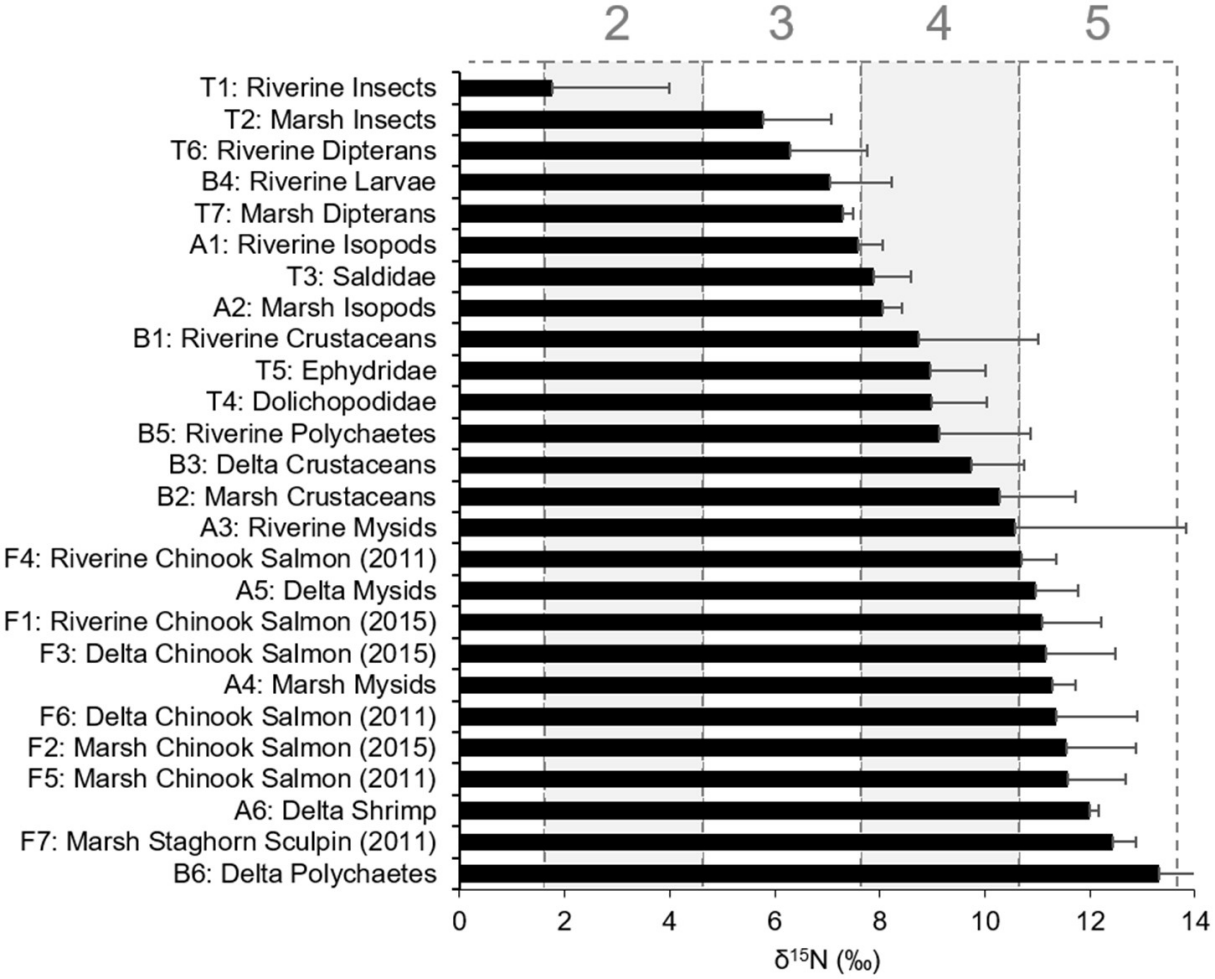
Consumer trophic levels. Nisqually River Delta consumer δ^15^N values and trophic levels (shaded rectangles numbered 2–5). Consumer groups and their associated taxa are listed in Table 2. Error bars represent ± 1 SD.

Food webs spanned two to four trophic levels (TL) and trophic “breadth” varied by habitat. The riverine food web, including invertebrate and fish consumers from freshwater and tidal-forested habitats, encompassed four TL (Fig 2). Riverine terrestrial insects such as hemipterans, hymenopterans, coleopterans, and lepidopterans had the lowest TL, representing δ^15^N_Baseline_ values (TL 2). Dipteran larvae and adults from the Chironomidae and Sciomyzidae families were in TL 3. Aquatic crustaceans such as the isopod *Gnorimosphaeroma oregonensis*, corophiid amphipods, and cumaceans were in TL 4, along with *Neanthes* polychaetes. The top consumer position in the riverine environment was occupied by the mysid *Neomysis mercedis* and juvenile Chinook salmon, which had higher δ^15^N values in 2015 than in 2011.

Marsh invertebrate and fish consumers were sampled in the transitional and emergent salt marsh habitats and spanned three TL (Fig 2). Insects (hemipterans, lepidopterans) and dipterans (Chironomidae, Muscidae, Tipulidae) were primary consumers in TL 3. Dipterans and hemipterans that typically feed on shoreline detritus such as Saldidae, Dolichopodidae, and Ephydridae occupied TL 4, along with isopods and other benthic crustaceans (corophiid amphipods and harpacticoid copepods). Top sampled consumers (TL 5) included *N*. *mercedis*, juvenile Chinook salmon (2011 and 2015), and sculpin.

The delta food web included consumers from the mudflat and eelgrass habitats and was comprised of benthic and pelagic taxa. Because delta primary producers tended to be more δ^15^N-enriched, crustaceans such as amphipods, harpacticoid copepods, cumaceans, and tanaids were in TL 4 (Fig 2). Barring allochthonous exchange from marsh and riverine invertebrates (e.g., drift insects), we did not observe any consumers in the delta that had a TL less than 4. Of the invertebrates and fish that were sampled, *N*. *mercedis*, juvenile Chinook salmon (2011 and 2015), *Crangon* shrimp, and polychaetes were at the top of the delta food web (TL 5; Table 2).

### Dietary composition of invertebrate consumers

**Fig 3:**
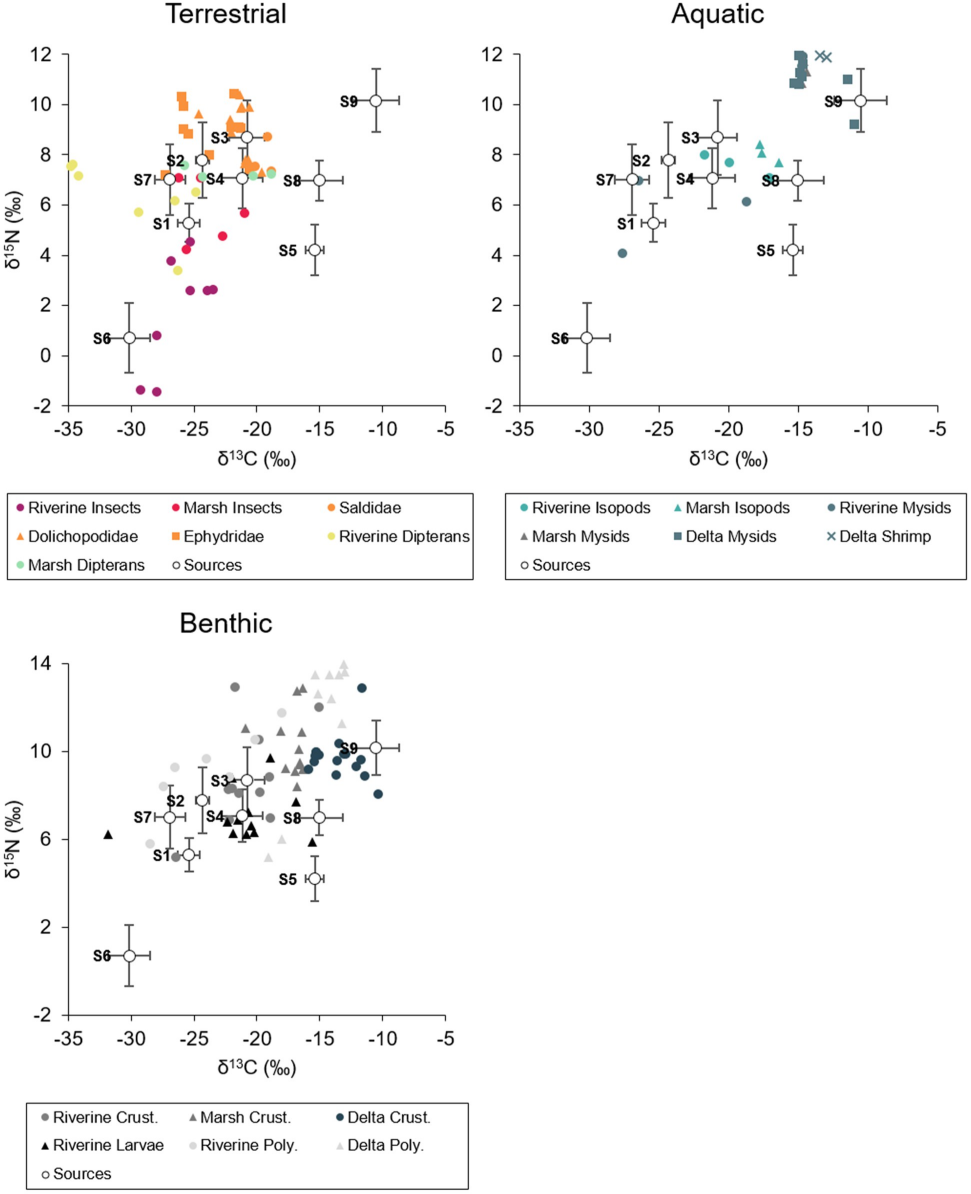
Biplots of Nisqually River Delta primary source and invertebrate consumer stable isotope signatures. Source labels match groupings in Table 1, but mean δ^13^C and δ^15^N values are adjusted for trophic fractionation. Error bars represent ± 1 SD.

**Fig 4:**
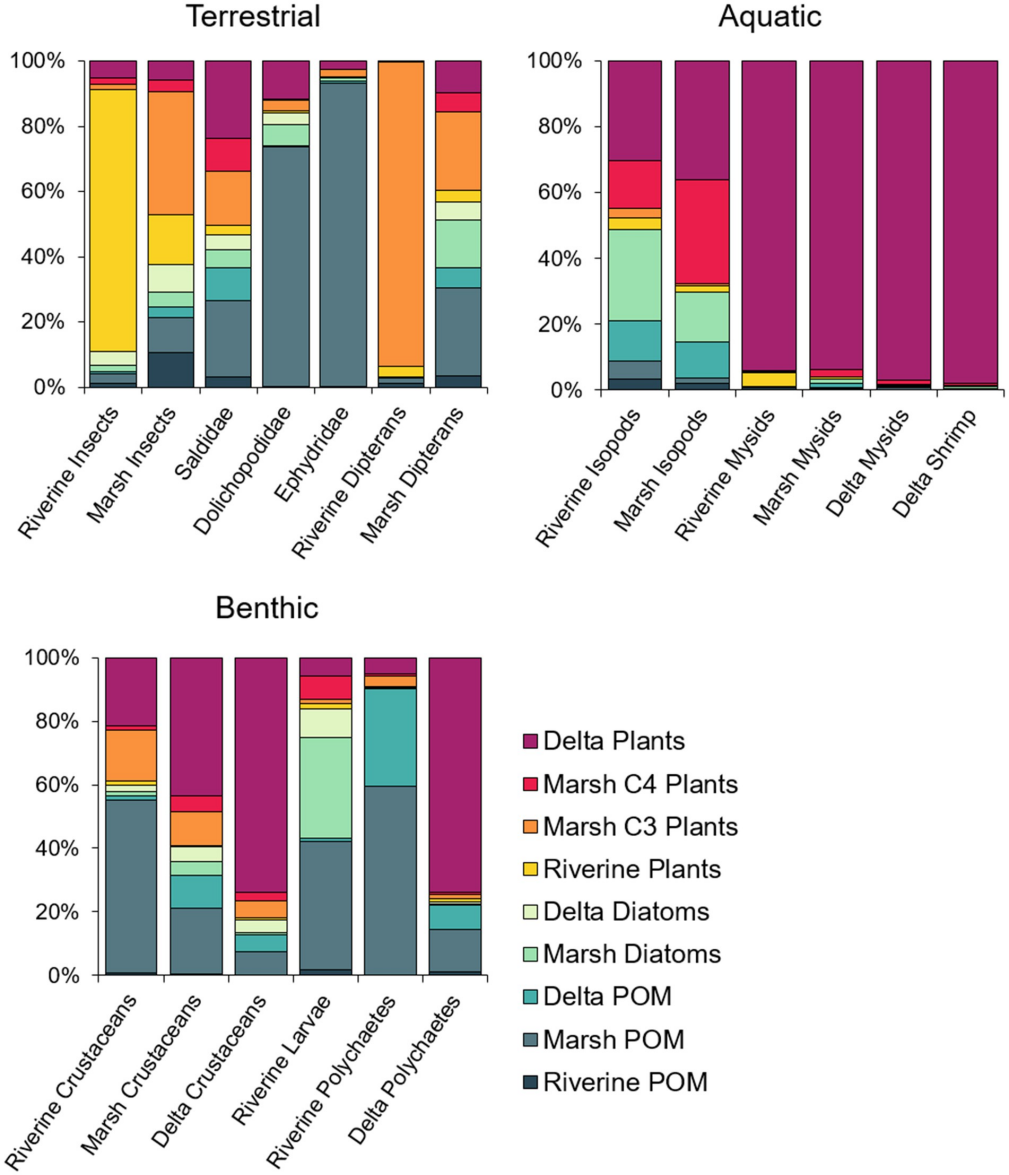
Percent contribution of primary sources to invertebrate consumer diets. The percent contribution of primary sources to invertebrate consumer diets in the Nisqually River Delta was predicted using a Bayesian mixing model of δ^13^C and δ^15^N isotopes. Bar plots show the estimated mean posterior probability. Colors correspond to source groupings in Fig 7.

**Fig 5:**
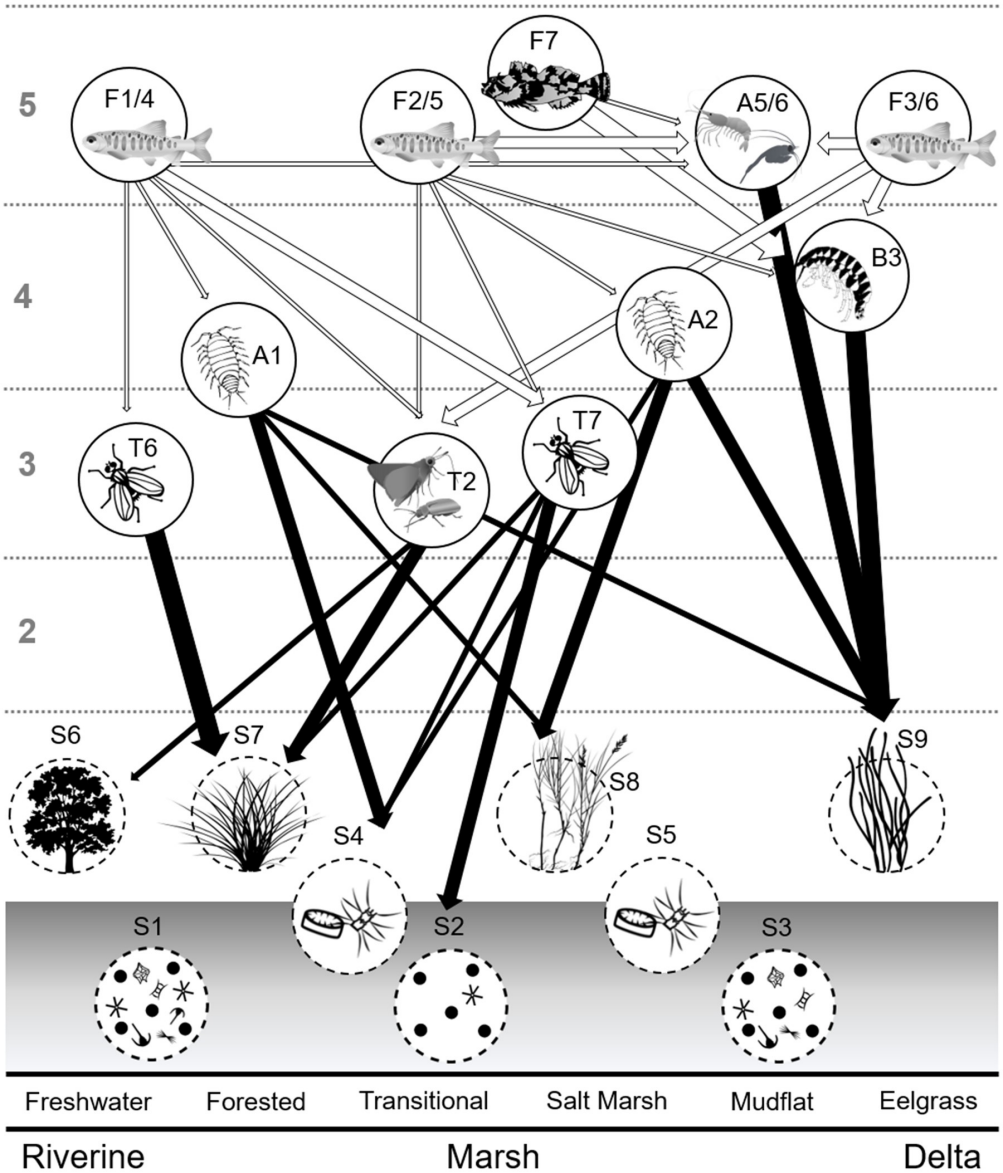
The Nisqually River Delta food web. The Nisqually River Delta food web was interpreted from a Bayesian mixing model of δ^13^C and δ^15^N. Alphanumeric labels correspond with the source groupings in Table 1 and consumer groupings in Table 2. Thin arrows indicate a 10–24% proportion contribution to diet, medium arrows indicate a 25–50% proportion contribution to diet, and thick arrows indicate a >50% contribution to diet. White arrows show the diets of fish consumers, while black arrows show the diets of invertebrate consumers. Gray numbers along the left-hand side of the figure designate consumer trophic levels based on δ^15^N values. Primary sources and consumers are visualized along a gradient of delta habitat types with salinity increasing from left to right.

We evaluated the diets of invertebrate consumers from the terrestrial, aquatic, and benthic environmental strata separately. For terrestrial invertebrates, we analyzed seven consumer groups which varied widely in their stable isotope concentrations and predicted dietary composition (PERMANOVA; *F*_*6*,*43*_ = 8.85, *p* < 0.001; Fig 3, Table 3). Riverine and marsh terrestrial insects such as coleopterans, hemipterans, hymenopterans, and lepidopterans were most likely to consume organic matter from vascular plants. Although they did not differ significantly in δ^13^C, δ^15^N, and δ^34^S, output from the SIMM predicted that the contribution of riverine plants was greatest for riverine insects, while the contribution of marsh C3 plants was greatest for marsh insects (Fig 4). Riverine dipterans also appeared to derive most of their energy from marsh C3 plants, resulting in potential marsh subsidies to the riverine environment (Fig 5). In contrast, marsh dipterans (including Dolichopodidae, Ephydridae, and other taxa) derived most of their energy from marsh POM, which had the lowest chlorophyll *a* concentrations and a C:N ratio > 9, and thus was more likely to include detrital organic matter.

**Table 3:** Pairwise comparisons of invertebrate consumer isotope signatures.

Consumer Group	*p*-value (PERMANOVA)					
	**Terr 2**	**Terr 3**	**Terr 4**	**Terr 5**	**Terr 6**	**Terr 7**
Terr 1: Riverine Insects	0.069	**0.045**	**< 0.001**	**< 0.001**	**< 0.001**	**< 0.001**
Terr 2: Marsh Insects	-	0.177	**< 0.001**	**< 0.001**	0.175	0.297
Terr 3: Saldidae	-	-	**0.032**	0.078	**0.032**	0.092
Terr 4: Dolichopodidae	-	-	-	**< 0.001**	**< 0.001**	**< 0.001**
Terr 5: Ephydridae	-	-	-	-	**< 0.001**	**0.032**
Terr 6: Riverine Dipterans	-	-	-	-	-	0.092
Terr 7: Marsh Dipterans	-	-	-	-	-	-
	**Aq 2**	**Aq 3**	**Aq 4**	**Aq 5**	**Aq 6**	
Aq 1: Riverine Isopods	**< 0.001**	**< 0.001**	**< 0.001**	**< 0.001**	0.114	
Aq 2: Marsh Isopods	-	**< 0.001**	0.167	**< 0.001**	0.114	
Aq 3: Riverine Mysids	-	-	**< 0.001**	**< 0.001**	**< 0.001**	
Aq 4: Marsh Mysids	-	-	-	0.114	0.138	
Aq 5: Delta Mysids	-	-	-	-	**< 0.001**	
Aq 6: Delta Shrimp	-	-	-	-	-	
	**Ben 2**	**Ben 3**	**Ben 4**	**Ben 5**	**Ben 6**	
Ben 1: Riverine Crustaceans	**< 0.001**	**< 0.001**	**0.042**	0.139	**< 0.001**	
Ben 2: Marsh Crustaceans	-	**< 0.001**	**< 0.001**	**< 0.001**	**< 0.001**	
Ben 3: Delta Crustaceans	-	-	**< 0.001**	**< 0.001**	**< 0.001**	
Ben 4: Riverine Larvae	-	-	-	**< 0.001**	**< 0.001**	
Ben 5: Riverine Polychaetes	-	-	-	-	**< 0.001**	
Ben 6: Delta Polychaetes	-	-	-	-	-	

Pairwise, among-group comparisons of terrestrial, aquatic, and benthic invertebrate consumers’ δ^13^C, δ^15^N, and δ^34^S signatures in the Nisqually River Delta. We used a PERMANOVA test with 1,000 model permutations to conduct multivariate analyses, where a cutoff value of *p* < 0.05 indicated that consumer groups were significantly different from each another. Groups that are *not* significantly different are highlighted in bold font.

Aquatic invertebrates also differed significantly in their δ^13^C, δ^15^N, and δ^34^S concentrations, although marsh and delta *N*. *mercedis* and *Crangon* shrimp were highly similar, as were riverine and marsh isopods (*F*_*5*,*25*_ = 2.74, *p* = 0.032; Fig 3, Table 3). Output from the SIMM indicated that up to 100% of mysid and shrimp diets consisted of delta plants (Figs 4 and 5). Riverine mysids also consumed primarily delta plants, although three outlier samples included potential contributions from riverine POM and riverine plants. Isopod diets were more variable, consisting of a mixture of marsh diatoms, marsh C4 plants, delta POM, and delta plants.

Marsh POM and delta plants were predicted to be the most important contributors to benthic invertebrate diets. Riverine insect larvae, amphipods, and polychaetes all derived at least 40% of their energy on average from marsh POM, even though their δ^13^C, δ^15^N, and δ^34^S values differed significantly (*F*_*5*,*73*_ = 20.11, *p* < 0.001; Figs 3 and 4, Table 3). For riverine insect larvae, the SIMM indicated that marsh diatoms also comprised roughly 32% of their dietary composition, while polychaetes were influenced by delta POM (31%). Not surprisingly, marsh and delta crustaceans (amphipods, copepods, cumaceans, and tanaids) and delta polychaetes appeared to consume more delta plants, with average contributions of up to 75% (Fig 5). Marsh crustaceans were also somewhat influenced by marsh POM (21%), demonstrating that the influence of autochthonous primary producers shifted along a salinity gradient.

### Dietary composition of fish consumers

**Fig 6:**
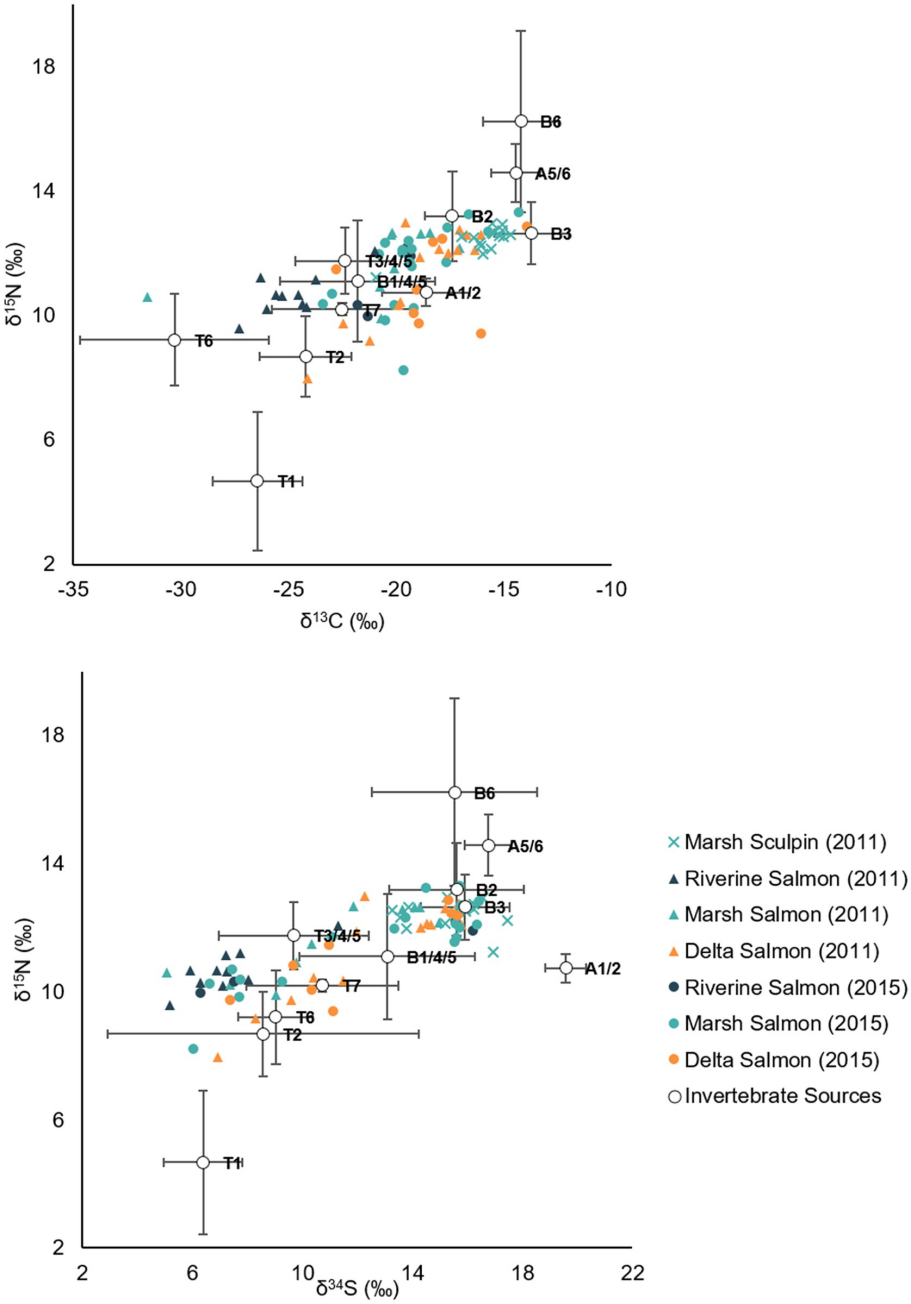
Biplots of Nisqually River Delta invertebrate and fish stable isotope signatures. To minimize variance and source overlap, invertebrates (Table 2) have been consolidated into coarser source groupings as follows: “riverine insects” (T1), “marsh insects” (T2), “riverine/marsh shoreflies and shorebugs” (T3/4/5), “riverine dipterans” (T6), “marsh dipterans” (T7), “riverine/marsh isopods” (A1/2), “delta mysids and shrimp” (A5/6), “riverine benthics” (B1/4/5), “marsh crustaceans” (B2), “delta crustaceans” (B3), and “delta polychaetes” (B6). Mean δ^13^C, δ^15^N, and δ^34^S values are adjusted for trophic fractionation based on estimates from Davis et al. [68]. Error bars represent ± 1 SD.

**Fig 7:**
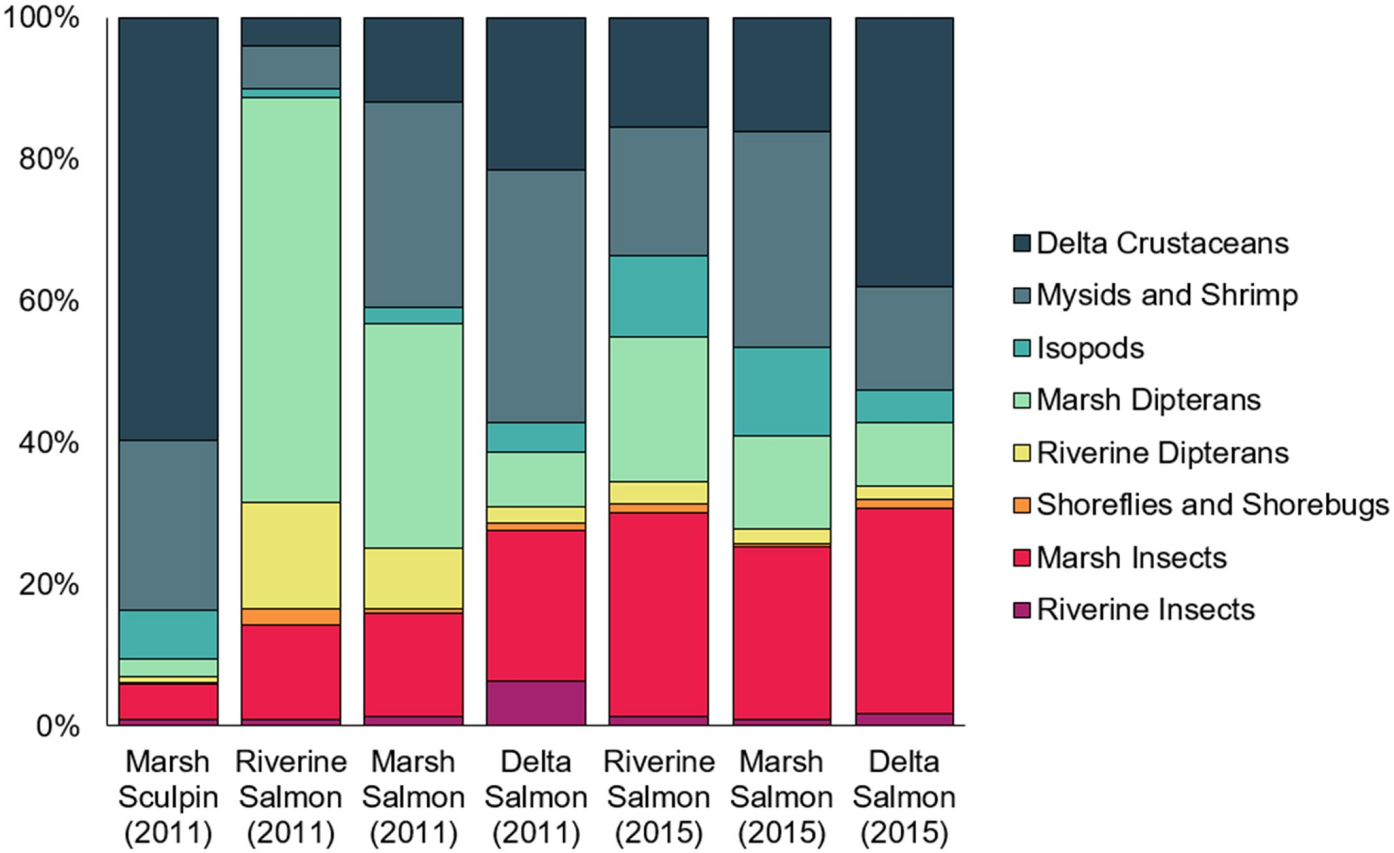
Percent contribution of invertebrate sources to fish diets. The percent contribution of invertebrate sources to pelagic (Chinook salmon) and demersal (sculpin) fish diets in the Nisqually River Delta was predicted using a Bayesian mixing model of δ^13^C, δ^15^N, and δ^34^S isotopes. Bar plots show the estimated mean posterior probability. Sources that contributed to <1% of all diets have been excluded, including riverine benthics (B1/4/5 in Table 2), marsh crustaceans (B2), and delta polychaetes (B6). Colors correspond to source groupings in Fig 3 (except for those sources that were excluded).

Pelagic and demersal fish differed in stable isotope composition such that sculpin were more enriched in δ^13^C, δ^15^N, and δ^34^S, even when compared to juvenile Chinook salmon that were captured in the delta’s nearshore intertidal zone (Table 2, Fig 6). Sculpin were predicted to have the greatest proportion of delta crustaceans in their diets (amphipods, copepods, cumaceans, and tanaids; 59%), with additional inputs from delta mysids and shrimp (24%; Figs 5 and 7).

For juvenile Chinook salmon, the contribution of prey sourced from riverine and marsh habitats and from the terrestrial stratum decreased from river to sea in both 2015 and 2011; however the shift was clearer in 2011 (Fig 7; also see Woo et al. [15]). In 2011, the total contribution from riverine and marsh insects and dipterans was 88% for Chinook salmon captured in the river, 56% for Chinook salmon captured in the marsh, and 39% for Chinook salmon captured in the delta. In 2015, the total contribution from riverine and marsh insects and dipterans was 51% for Chinook salmon captured in the river, 42% for Chinook salmon captured in the marsh, and 43% for Chinook salmon captured in the delta. The SIMM indicated that most of the terrestrially-sourced prey in Chinook salmon diets were adult insects and dipterans from the emergent marsh, even when fish had been caught upstream in the river. The model also showed that once fish began rearing in the marsh and delta, mysids, shrimp, and other crustaceans frequently served as alternative prey resources (Fig 5).

**Fig 8:**
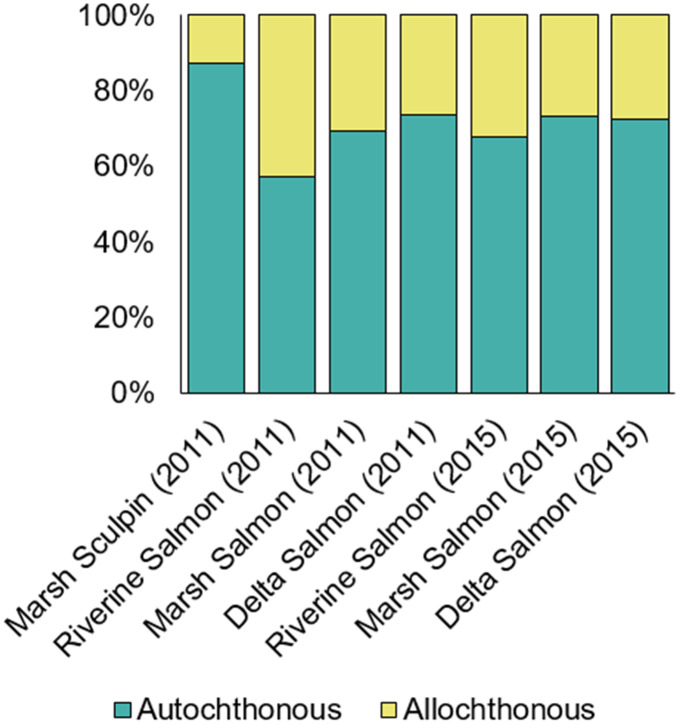
Estimated proportion of allochthonous and autochthonous organic matter contributions to Nisqually River Delta fish diets. Allochthonous organic matter includes riverine plants and marsh C3 and C4 plants. Autochthonous organic matter includes riverine, marsh, and delta POM, marsh and delta diatoms, and delta plants.

The proportional contribution of allochthonous and autochthonous organic matter differed between salmon and sculpin. Allochthonous primary producers such as riverine plants and marsh C3 and C4 plants comprised only 13% of sculpin diets, while salmon diets were made up of 26–43% of allochthonous material depending on year and habitat of capture (Fig 8). For salmon, there was a river-to-ocean signal in the degree of allochthonous influence, which was more detectable in 2011. The proportional contribution of allochthonous organic matter decreased from 43% to 26% in 2011, but in 2015 it only decreased from 32% to 27%. The discrepancy among years and the reduction of allochthonous influence in the river in 2015 appears to be driven by reduced consumption of marsh insects in favor of delta crustaceans, mysids, and shrimp, indicating that salmon in 2015 spent more time rearing in delta habitat.

## Discussion

Results from the Nisqually River Delta highlight how allochthonous and autochthonous organic matter from throughout an integrated habitat mosaic supports the estuarine food web. Delta plants such as marine macroalgae and eelgrass appear to be important base components of pelagic and demersal food webs in the NRD, contributing a substantial portion of energy (in many cases >50%) to pelagic and benthic crustaceans and their fish predators. Allochthonous marsh C3 plants like sedges, rushes, and halophilic salt marsh species were the second most important source of organic matter, especially for juvenile Chinook salmon and their terrestrial insect prey. Marsh C3 plant matter made its way into the pelagic food web via indirect pathways whereby adult insects from riverine and marsh habitats fell or were washed into the water column from the surrounding shoreline. The lack of evidence for direct (as opposed to consumptive) contributions of allochthonous detritus may indicate selection for specific prey items by juvenile Chinook salmon and/or weakened detrital delivery pathways between the terrestrial and aquatic environments. Terrestrial inputs have likely been disconnected by decades of habitat degradation with only recent recovery from restoration activities in the NRD. These findings are discussed in greater detail below.

### Pelagic and demersal food web structure

There were clear differences in the structure and composition of pelagic and demersal food webs in the NRD, even in marsh habitats where sculpin and juvenile Chinook salmon demonstrated spatiotemporal overlap. Not surprisingly, the SIMM found that sculpin were most likely to consume crustaceans that live in benthic substrates such as amphipods and harpacticoid copepods. These taxa comprised almost 60% of sculpin diets in 2011, while another 24% was comprised of pelagic crustaceans such as mysids and shrimp. These prey taxa relied almost exclusively on delta plants such as marine macroalgae and eelgrass, with marginal influence (less than 5–10%) of marsh POM and C3 vascular plants.

Marsh- and delta-rearing salmon also demonstrated strong preferences for delta crustaceans, mysids, and shrimp in both 2011 and 2015; however, the SIMM indicated there were substantial dietary contributions from marsh insects like hemipterans, which mostly consumed riverine and marsh C3 plant matter. It is likely that juvenile salmons’ generalist feeding strategy was supported by foraging across terrestrial, pelagic, and benthic environmental strata and habitat types [18, 85, 86]. The salmon food web was consistently influenced by marsh C3 plants via indirect pathways, whereby juvenile salmon consumed dipterans and insects that fell into the water column. This was true for fish captured in riverine, marsh, and delta habitats, demonstrating how emergent transitional and salt marshes can subsidize multiple, interconnected estuarine habitat types [15, 17, 87]. The relative importance of these marsh subsidies varied ontogenetically as juvenile salmon moved from river to sea, with this gradient being more obvious in 2011 than in 2015. Fish captured in riverine habitats derived most of their energy from terrestrial insects (almost 90% in 2011 and about 50% in 2015), as did fish captured in marsh habitats, albeit to a lesser degree. Delta-rearing salmon in both years were less reliant on adult insect prey and more likely to consume benthic amphipods and pelagic crustaceans, which showed strong signals for delta plants such as eelgrass and ulvoid algae. These findings are supported by stomach content data that were collected concurrently with fish tissue samples [18].

Ultimately, out-migrating juvenile salmon rely on multiple habitat types throughout the estuarine gradient, which is reflective of their diverse foraging habitats in the NRD and in other estuarine systems [18, 88]. Dietary composition is influenced both by the amount of time fish spend foraging in each estuarine habitat type and the movement of primary producers and prey items in and out of the system. We observed evidence for linkages among environmental strata and across a salinity gradient. Vascular plants contributed to the diets of marsh-rearing insects and detritivorous isopods, which in turn were eaten by juvenile salmon. Conversely, marsh- and delta-derived autochthonous organic matter such as phytoplankton, diatoms, algae, and eelgrass contributed considerably to the diets of insects with aquatic life stages and to some crustaceans and polychaetes that were sampled in riverine habitats. Proximity to marsh-adjacent habitat and other areas with high primary productivity and abundant prey resources can provide measurable benefits for juvenile salmon in terms of growth and survival [89–91]. Thus, this study joins others in underscoring the importance of connectivity, and conservation of multiple estuarine habitat types for salmon and other estuary-dependent fishes.

### Contributions of allochthonous and autochthonous primary production

We examined the structure of NRD food webs by quantifying allochthonous and autochthonous sources of organic matter from throughout an estuarine salinity gradient and across terrestrial, pelagic, and benthic environmental strata. For the sake of our study, allochthonous sources of organic matter were of terrestrial (external) origin and autochthonous sources were of aquatic (internal) origin [21, 22].

Relative proportions of allochthonous and autochthonous contributions differed between pelagic and demersal food webs. Allochthonous contributions to demersal sculpin diets were less than 13%, while allochthonous contributions to pelagic juvenile Chinook salmon diets ranged from 26–43% and were generally greater in riverine and marsh habitats. Other studies have observed similar patterns driven by spatial habitat use, foraging preferences, and ontogeny. Botto et al. [32] found that the influence of terrestrial plant matter on the estuarine food web of a South American river depended on turbidity and salinity. Terrestrial inputs were generally detrital in nature in this system, whereas in the NRD, allochthonous organic matter was introduced via indirect consumptive pathways of fresh, rather than detrital, organic matter. Findings from Drexler et al. [19], which showed substantial contributions of marsh plant detritus from nearby mature marshes in the sediments of sparsely-vegetated restoration marsh, would suggest that plant detritus is being delivered to the aquatic environment, but that detritivorous primary consumers are not accessing this food source. The pathways by which allochthonous organic matter is delivered to the estuarine food web can vary substantially within and among systems. In Arctic coastal lagoons, McMahon et al. [34] and Dunton et al. [92] observed substantial contributions of terrestrial organic matter to the food web via direct and indirect pathways, especially in river-adjacent areas. In the San Francisco Bay Estuary, the influence of terrestrial vascular plant matter was dependent on seasonal shifts in freshwater flows, proximity to marsh habitats, and species foraging strategy [17, 20].

We also observed differences in the way environmental factors like stream flow can affect food web structure within the same system because we sampled juvenile Chinook salmon in 2011, a climatologically average water year, and 2015, an extreme drought year. Allochthonous contributions to the pelagic food web were lower in 2015 than in 2011, and the river-to-sea habitat gradient was less obvious. This reflects how juvenile salmon move among habitats to avoid elevated temperatures and lower water levels in the river and estuary [16, 91, 93, 94]. Furthermore, it supports existing literature that shows how drought conditions can reduce inputs of terrestrial organic matter from the surrounding marsh into the estuary by shifting vascular plant biomass availability and prey community structure [20, 95–97].

Stable isotope analysis indicated that autochthonous organic matter, particularly macroalgae and eelgrass from the nearshore intertidal zone, mostly supported the base of the NRD food web; however, estimates of allochthonous influence in both 2011 and 2015 were likely conservative. This is because we could not accurately determine the relative composition of riverine, marsh, or delta POM. Particulate organic matter is made up of various mixtures of phytoplankton, organic detritus of terrestrial and marine origin, and inorganic sediments [24]. Consequently, POM can be considered a mixture of both autochthonous and allochthonous organic matter sources. We used chlorophyll *a* and pheophytin concentrations, C:N ratios, and carbon isotope signatures to estimate the relative influence of autochthonous phytoplankton for each source group [78–80]. For juvenile salmon caught in riverine and marsh habitats, marsh POM was determined to be a substantial contributor to the pelagic food web, mostly via the consumption of marsh dipterans like chironomids, which have aquatic larval stages. We classified marsh POM as an autochthonous organic matter source, but chlorophyll *a* and pheophytin concentrations in marsh POM were relatively low, the C:N ratio of POM samples was slightly > 9, and carbon signatures were more depleted than the -22 to -20‰ range that would be expected for phytoplankton. This would suggest that marsh POM contained some terrestrial detritus, and that inputs of allochthonous organic matter were likely underestimated for the pelagic (salmon) food web.

### Analytical limitations

Bayesian SIMMs are a robust method of evaluating food web linkages across multiple species, trophic levels, and systems of interest; however, they are not without limitations. To accurately determine source contributions to consumer diets, sources must be sufficiently separated in *n*-dimensional isotopic space. Separation can be achieved by grouping sources with similar biological roles and isotopic signatures, omitting sources that do not appear to be biologically meaningful, or by analyzing additional isotopes [98–100]. For this study, we were unable to obtain sulfur isotope signatures for POM or diatoms, so we could only run a two-isotope SIMM with carbon and nitrogen when evaluating the relative contributions of different organic matter sources among invertebrate consumers. Nevertheless, we were able to combine and omit sources such that they were adequately separated in two-dimensional space. Most invertebrate consumers fell within the source “polygon” with the notable exception of mysids and shrimp which were enriched in δ^15^N. The relatively high trophic position of these taxa suggests that, although they were treated as primary consumers in the SIMM, they are in fact omnivorous (feeding on zooplankton and phytoplankton) or carnivorous. This is consistent with what is known about the ecological roles of these crustacean species in Puget Sound and along the North American Pacific coast [101–103].

### Management implications

This study contributes to a foundational understanding of how environmental conditions and ecological connectivity influence primary productivity, prey productivity, and the directionality of energy flows in estuarine systems. It demonstrates how estuarine food webs transcend stratification of environmental resources and established habitat margins. We observed evidence for linkages across environmental strata and along a salinity gradient. Autochthonous plant matter from the nearshore (delta) intertidal zone strongly influenced the diets of sculpin and juvenile salmon captured throughout the entire habitat mosaic, including in riverine and marsh habitats. Similarly, allochthonous marsh C3 plants contributed to the food web via the consumption of adult insects and dipterans. Connectivity among the estuarine habitat mosaic and between terrestrial and aquatic environments can subsidize food webs for pelagic fish species such as (ESA)-listed juvenile salmon. This has implications for management because it suggests that habitat restoration and enhancement actions for such species will be most effective if they can encourage the longitudinal and lateral delivery of riverine and marsh allochthonous inputs and improve the connectivity of the terrestrial-aquatic interface. Studies that further elucidate food web structure, connectivity and resilience associated with climatic changes can provide further insights for management agencies.

In the NRD, allochthonous inputs were limited to indirect pathways, whereby adult insects fell or were washed into the water column and eaten by fish. There was little evidence for the direct consumption of terrestrial organic matter by pelagic and benthic invertebrate species other than isopods (although, as mentioned above, we may have underestimated the contribution of particulate detrital matter originating from marsh and riparian landscapes). Additionally, evidence for riverine influence on pelagic and demersal food webs was weak, even though riverine organic matter has been found to contribute substantially to local restoring marsh sediments [19]. This suggests, in addition to the sparsely vegetated 2009 restoration area, that the NRD food web may still be in a state of recovery following decades of habitat degradation and major restoration efforts in 2009. Further, it indicates that ecological processes to restore food web functions may occur on longer timescales than the restoration of structural features [72]. Continued monitoring and evaluation, including the use of stable isotopes, would allow managers to track development through time and enact adaptive management measures such as enhanced longitudinal connectivity and riparian habitat quality.

Allochthonous marsh subsidies provide ecosystem benefits that go well beyond food web support. For example, there is evidence that marsh and riverine organic matter can improve system-wide stability by promoting sediment accretion in addition to inorganic mineral sediments [19, 104–106]. Estuaries also provide distinct carbon sequestration benefits [15, 71, 107], with various mechanisms promoting effective sequestration among different habitat types [108, 109]. Environmental characteristics such as the type, quality, and amount of riparian and marsh vegetation, tidal and riverine fluxes, and linkages among vegetated habitats affect the way that terrestrial organic matter moves among habitats and between terrestrial and aquatic strata [6–10]. Consequently, it is not just the quantity and quality of riparian and marsh habitats, but their connectivity to the broader ecosystem mosaic that determines how allochthonous subsidies provide ecosystem benefits in estuaries and other aquatic ecosystems.
