# Correction: Allochthonous marsh subsidies enhances food web productivity in an estuary and its surrounding ecosystem mosaic

**DOI:** 10.1371/journal.pone.0331309

**Published:** 2025-09-03

**Authors:** Melanie J. Davis, Isa Woo, Susan E. W. De La Cruz, Christopher S. Ellings, Sayre Hodgson, Glynnis Nakai

The word “enhance” is misspelled in the article title. The correct title is: Allochthonous marsh subsidies enhance food web productivity in an estuary and its surrounding ecosystem mosaic. The correct citation is: Davis MJ, Woo I, De La Cruz SEW, Ellings CS, Hodgson S, Nakai G (2024) Allochthonous marsh subsidies enhance food web productivity in an estuary and its surrounding ecosystem mosaic. PLoS ONE 19(2): e0296836. https://doi.org/10.1371/journal.pone.0296836
